# Investigation of cardiovascular protective effect of Shenmai injection by network pharmacology and pharmacological evaluation

**DOI:** 10.1186/s12906-020-02905-8

**Published:** 2020-04-15

**Authors:** Lin Li, Dongli Yang, Jinghao Li, Lu Niu, Ye Chen, Xin Zhao, Patrick Kwabena Oduro, Chun Wei, Zongpei Xu, Qilong Wang, Yuhong Li

**Affiliations:** 1 Institute of Traditional Chinese Medicine, Tianjin University of Traditional Chinese Medicine, Tianjin, 301617 China; 2 Key Laboratory of Pharmacology of Traditional Chinese Medical Formulae, Ministry of Education, Tianjin University of Traditional Chinese Medicine, Tianjin, 301617 China; 3 Tianjin Medical University Cancer Hospital, Tianjin, 300060 China

**Keywords:** Network pharmacology, Shenmai injection, Doxorubicin, Cardiotoxicity, PI3K/Akt signaling pathway

## Abstract

**Background:**

Shenmai injection (SMI) has been used in the treatment of cardiovascular disease (CVD), such as heart failure, myocardial ischemia and coronary heart disease. It has been found to have efficacy on doxorubicin (DOX)-induced cardiomyopathy. The aims of this study were to explore the underlying molecular mechanisms of SMI treatment on CVD by using network pharmacology and its protective effect on DOX-induced cardiotoxicity by in vitro and in vivo experiment based on network pharmacology prediction.

**Methods:**

Network pharmacology method was used to reveal the relationship between ingredient-target-disease and function-pathway of SMI on the treatment of CVD. Chemical ingredients of SMI were collected form TCMSP, BATMAN-TCM and HIT Database. Drugbank, DisGeNET and OMIM Database were used to obtain potential targets for CVD. Networks were visualized utilizing Cytoscape software, and the enrichment analysis was performed using IPA system. Finally, cardioprotective effects and predictive mechanism confirmation of SMI were investigated in H9c2 rat cardiomyocytes and DOX-injured C57BL/6 mice.

**Results:**

An ingredient-target-disease & function-pathway network demonstrated that 28 ingredients derived from SMI modulated 132 common targets shared by SMI and CVD. The analysis of diseases & functions, top pathways and upstream regulators indicated that the cardioprotective effects of SMI might be associated with 28 potential ingredients, which regulated the 132 targets in cardiovascular disease through regulation of G protein-coupled receptor signaling. In DOX-injured H9c2 cardiomyocytes, SMI increased cardiomyocytes viability, prevented cell apoptosis and increased PI3K and p-Akt expression. This protective effect was markedly weakened by PI3K inhibitor LY294002. In DOX-treated mice, SMI treatment improved cardiac function, including enhancement of ejection fraction and fractional shortening.

**Conclusions:**

Collectively, the protective effects of SMI on DOX-induced cardiotoxicity are possibly related to the activation of the PI3K/Akt pathway, as the downstream of G protein-coupled receptor signaling pathway.

## Background

Doxorubicin (DOX) is a widely used chemotherapeutic drug in the treatment of human solid and hematogenous malignancies. However, it has cumulative dose-dependent cardiotoxic effects, which can lead to cardiac dysfunction, cardiomyopathy, and even severe heart failure [1]. When the cumulative dose of DOX is 400 mg/m^2^, the risk of heart failure is at an average of 5%. This risk increased exponentially at higher doses of DOX [2]. Moreover, the incidence of subclinical and overt cardiotoxicity in cancer patients treated with DOX after 9 years follow-up was 17.9 and 6.3%, respectively [3]. Therefore, with the increasing population of cancer survivors treated with DOX, its cardiotoxicity arises a wide concern. Dexrazoxane (DEX) is an established cardio-protectant, with a protective effect of the heart from DOX. DEX, the only Food and Drug Administration (FDA) approved-medicine that is used in combination with DOX. However, evidence showed that DEX aggravates bone marrow suppression induced by chemotherapeutic drugs [4]. Therefore, it is urgent to find cardioprotective medicines with both high efficiency and low toxicity as the combined medication with chemotherapeutic drugs.

Traditional Chinese Medicine (TCM), which featured as having “multiple ingredients and multiple targets”, has been used for treating complex diseases for decades [5]. Shenmai injection (SMI), composed of Ginseng Radix et Rhizoma Rubra (GR) and Ophiopogonis Radix (OR), is derived from Shengmaisan in Qianjin Yaofang. SMI has been approved by the China Food and Drug Administration (CFDA) for the treatment of chronic corpulmonale heart failure since 1995 [6]. Shreds of evidence have reported that the mechanisms of SMI in the treatment of CVD were related to improving the electrophysiological activity in hypertrophic rat myocardium [7], up-regulating nitric oxide level, increasing superoxide dismutase activity, decreasing endothelin-1 levels, and improving vascular endothelial-dependent vasodilation [8]. However, the underlying molecular mechanisms of the cardioprotective effect of SMI remain unexplored.

In 1999, SMI was first reported to be used in the treatment of DOX-induced cardiotoxicity [9]. Recent evidence showed that SMI prevented abnormal electrocardiogram, left ventricular ejection fraction (LVEF), and cardiac troponin (cTnT) caused by DOX [10]. Moreover, SMI can even reduce the incidence of bone marrow suppression caused by DEX [11, 12]. Although studies have reported that the protective effect of SMI on DOX-induced myocardial damage may be associated with scavenging free radical [13], relieving calcium overload [14], and protecting mitochondria function [15], the underlying molecular mechanisms has not been elucidated.

**Fig. 1:**
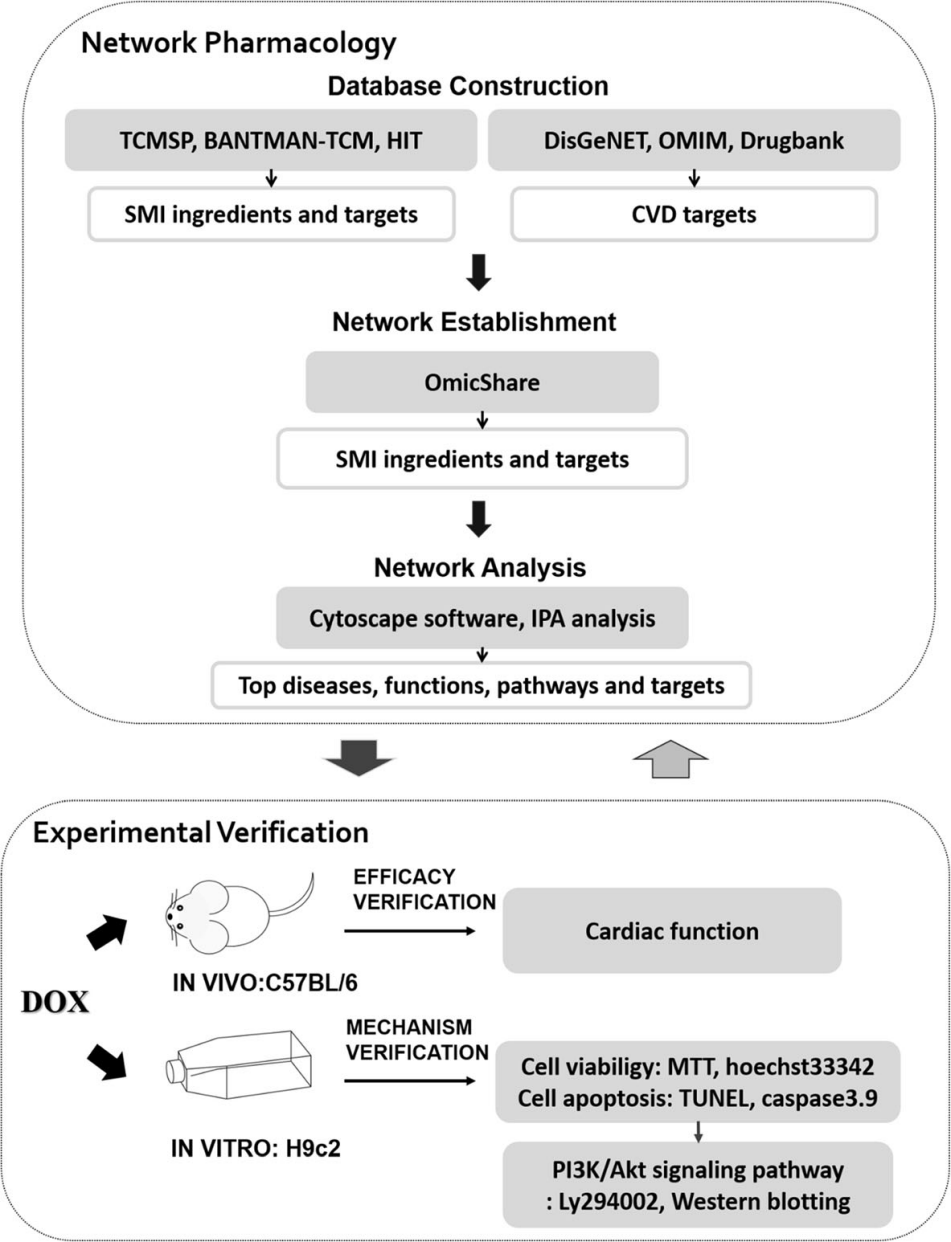
Workflow of the entire research

Network pharmacology has helped to unveil the complicated pharmacological mechanism of several TCM formulations by combining cheminformatics, bioinformatics, and network biology [16, 17]. SMI is a multi-component and multi-target agent, which exhibits cardioprotective efficacy through regulating molecular networks. Therefore, in this research, network pharmacology and experimental verification were combined to elucidate the potential mechanism of SMI on DOX-induced cardiotoxicity (Fig. 1). Further, H9c2 cells were used in vitro for mechanism verification. C57BL/6 mice injured by DOX were used in vitro for confirming cardioprotective efficacy of SMI.

## Methods

### SMI ingredients collection and targets fishing

The chemical ingredients of GR and OR were collected from the Traditional Chinese Medicine Systems Pharmacology (TCMSP) Database [18] (http://lsp.nwu.edu.cn/tcmsp.php) and the Bioinformatics Analysis Tool for Molecular mechANism of Traditional Chinese Medicine (BATMAN-TCM) Database [19] (http://bionet.ncpsb.org/batman-tcm/). Drug-likeness (DL) value, as a suggested criterion by TCMSP Database, was used for evaluation of absorption, distribution, metabolism and excretion (ADME) [20]. The ingredients were screened by DL value. When the DL value is ≥0.18, the ingredients were retained. Literature mining also supplemented the information from these databases. The protein targets of compounds from SMI were mainly collected from TCMSP and BATMAN-TCM. As a supplement, the Herbal Ingredients’ Targets (HIT) Database [21] (http://lifecenter.sgst.cn/hit/) was used for obtaining the validated targets. Due to the irregular naming of the retrieved target, UniProt Database [22] (http://www.uniprot.org/) was used for correcting all retrieved proteins to their official symbols by inputting protein names and restricting species to human beings.

### Cardiovascular disease genes collection

The potential targets for CVD were obtained from three resources: Drugbank Database [23] (https://www.drugbank.ca/), a database of gene-disease associations (DisGeNET) [24] (http://www.disgenet.org/), and Online Mendelian Inheritance in Man (OMIM) [25] (http://omim.org/).

### Identification of common targets of CVD and SMI

The OmicShare tool (http://www.omicshare.com/tools) was used for identification of the common targets of CVD and SMI. Briefly, the SMI and CVD associated targets dataset was constructed and uploaded into the Venn tool to identify common targets of CVD and SMI.

### Network construction and analysis

All the networks were visualized utilizing Cytoscape software [26] (Version 3.6.0). Network construction was performed as follows: (1) SMI targets PPI network; (2) CVD targets PPI network; (3) common targets of CVD and SMI PPI network; (4) SMI ingredient-target network; (5) target-disease & function-pathway network.

Enrichment Analysis was performed by the Ingenuity Pathway Analysis (IPA, version 01–12) system [27]. “Core analysis” module was used for obtaining top canonical pathways, upstream regulators, diseases and bio functions. The concerned canonical pathways were found by using “Overlay-Canonical Pathway” module. Then “Build-Diseases & Functions” module was performed to obtain the targets involved in diseases and bio functions. “Path designer” module was used for prettifying the network. The *P*-values represent the possibility that the molecules in the dataset were related to canonical pathways/diseases and bio functions/upstream regulators. The enrichment score of P-values was based on Fisher’s exact test.

### Cell culture and treatments

Rat embryonic ventricular myocardial cell line H9c2 was purchased from the American Type Cell Culture (ATCC, Manassas, VA). Cells were cultured as previously described [28]. Cells were pretreated with or without SMI (0.25% or 0.5% in DMEM) for 24 h. The pretreated cells were then treated with or without DOX (Sigma, St. Louis, MO, USA, 0.5 μM) for 24 h. Moreover, in some group, LY294002 (Selleckchem, USA, 10 μM) was added to cells for 1 h before DOX treatment.

### Cell viability measurements

Cell viability was examined by a cell counting kit 8 (CCK8, Dojindo Laboratories, Japan). After H9c2 cells were treated with DOX in the absence or presence of SMI or LY294002 at 37 °C in a 96-well plate, CCK8 solution (10 μL/100 μL medium) was added to the media for 3 h. The absorbance of the media at 450 nm was examined by a microplate reader (Infinitef50, Tecan, Switzerland).

### Nuclear staining with Hoechst 33342

Cells were incubated with DOX in the absence or presence of SMI or LY294002 at 37 °C. Then Hoechst 33342 (Sigma, St. Louis, MO, USA, 10 μg/mL) was applied for 15 min under dark condition to stain the nucleus of cells. Images were observed, and photomicrographs were taken with a fluorescence microscope (AX10, Carl Zeiss AG, Germany). Cells with a nucleus exhibiting brightly stained condensed chromatin, unclear fragments or apoptosis bodies were identified as damaged nucleus. The positive staining nucleus was calculated in 3 random fields (× 630). The damaged nucleus index was presented as the percentage of the number of positively stained nuclei to the number of all stained nuclei from 3 random fields in each group.

### TUNEL staining

The staining assay was measured using TUNEL kit (Roche, Mannheim, Germany). Apoptotic cells were counted in 3 random fields (× 100) by fluorescence microscope in each group.

### Western blotting

Total protein obtained from cardiac tissue and cardiomyocytes were quantified using bicinchoninic acid protein assay kits (CWBIO, Jiangsu, China). Equal amounts of proteins were separated by NUPAGE™ 10% Bis-Tris Gels and transferred onto a polyvinylidene fluoride (PVDF) membrane (Immobilon, USA). After being blocked, the membranes were incubated overnight at 4 °C with the corresponding primary antibody. After washing with TBST, the membranes were incubated for 1.5 h with secondary antibodies. All antibodies were purchased from Cell Signaling Technology (Danvers, MA, USA). The immunoblots were detected by western chemiluminescent HRP substrate (Immobilon, USA) and Amersham Imager 600 (GE Healthcare Bio-Sciences AB, Japan).

### Animal study

Male C57BL/6 mice weighing 22–25 g were purchased from Beijing Vital River Laboratory Animal Technology Co., Ltd. (certificate number: SCXK(JING)20,160,006). All animal experiments were carried out according to the National Institute of Health Guide for the Care and Use of Laboratory Animals, and were approved by the Animal Care and Use Committee of Tianjin University of Traditional Chinese Medicine. Mice were given free access to water and normal food under a 12-h light-dark cycle and specific pathogen-free conditions.

DOX (Shenzhen Main Luck Pharmaceuticals, Shenzhen, China) was dissolved in normal saline. Mice were randomly divided into 4 groups: normal control group (CON, *n* = 10), SMI control group (CON+SMI, n = 10), DOX injury group (DOX, *n* = 26) and SMI pretreatment group (DOX + SMI, *n* = 25). Due to the possibility of DOX-induced death, the number of mice in DOX injured-group was higher than the normal group, i.e., n = 26 and n = 10, respectively. Mice received a daily intraperitoneal (i.p.) injection of DOX (3 mg/kg/day for 5 days, i.p.) to obtain a cumulative dose of 15 mg/kg. SMI (Chia Tai Qingchunbao Pharmaceutical, Hangzhou, China, 2.5 ml/kg/day for 8 days, i.p.) was given 3 days before administration of DOX. Control mice received an intraperitoneal injection of normal saline. After 24 h of the last dose of DOX, body weight was recorded, and left ventricular function was evaluated. At the end of the experiment, all animals were anaesthetized with tribromoethanol solution (1 g 2,2,2-tribromoethanol dissolved in 0.625 mL 2-methyl-2-butanol and diluted to 20 mg/mL with normal saline, 0.1 mL/10 g body weight, i.p.). When mice were unconscious, blood was sampled from their orbital vein, and after which, they were euthanized by cervical dislocation, for immediate heart collection. Heart weight and tibial length were measured.

### Echocardiographic assessment

Echocardiography was performed using a Vevo 2100 Imaging System (Visual Sonics, Toronto, Canada). The mice were inhaled with isoflurane (1% oxygen plus 2–5% isoflurane) to induce anesthesia. Two-dimensional image obtained in the parasternal long-axis views. The percentage of fractional shortening (FS, %), ejection fraction (EF, %) and left ventricular posterior wall thickness (LVPW) were determined from the M-mode recordings.

### Assessment of serum markers of cardiac injury

Creatine kinase (CK), creatine kinase-MB isoenzyme (CK-MB) and lactate dehydrogenase (LDH) activities were measured using commercial kits (BIOSINO, Beijing, China) and automatic biochemical analyzer (Thermo Fisher Scientific, NY, USA).

### Statistical analysis

Data were expressed as mean ± SD. Statistical evaluations were performed by one-way analysis of variance (ANOVA) followed by LSD Method and Dunnett’s C test, using SPSS software (Version 24.0). Values of *P* < 0.05 were considered a statistically significant difference.

## Results

### Identification of common targets of CVD and SMI

**Fig. 2:**
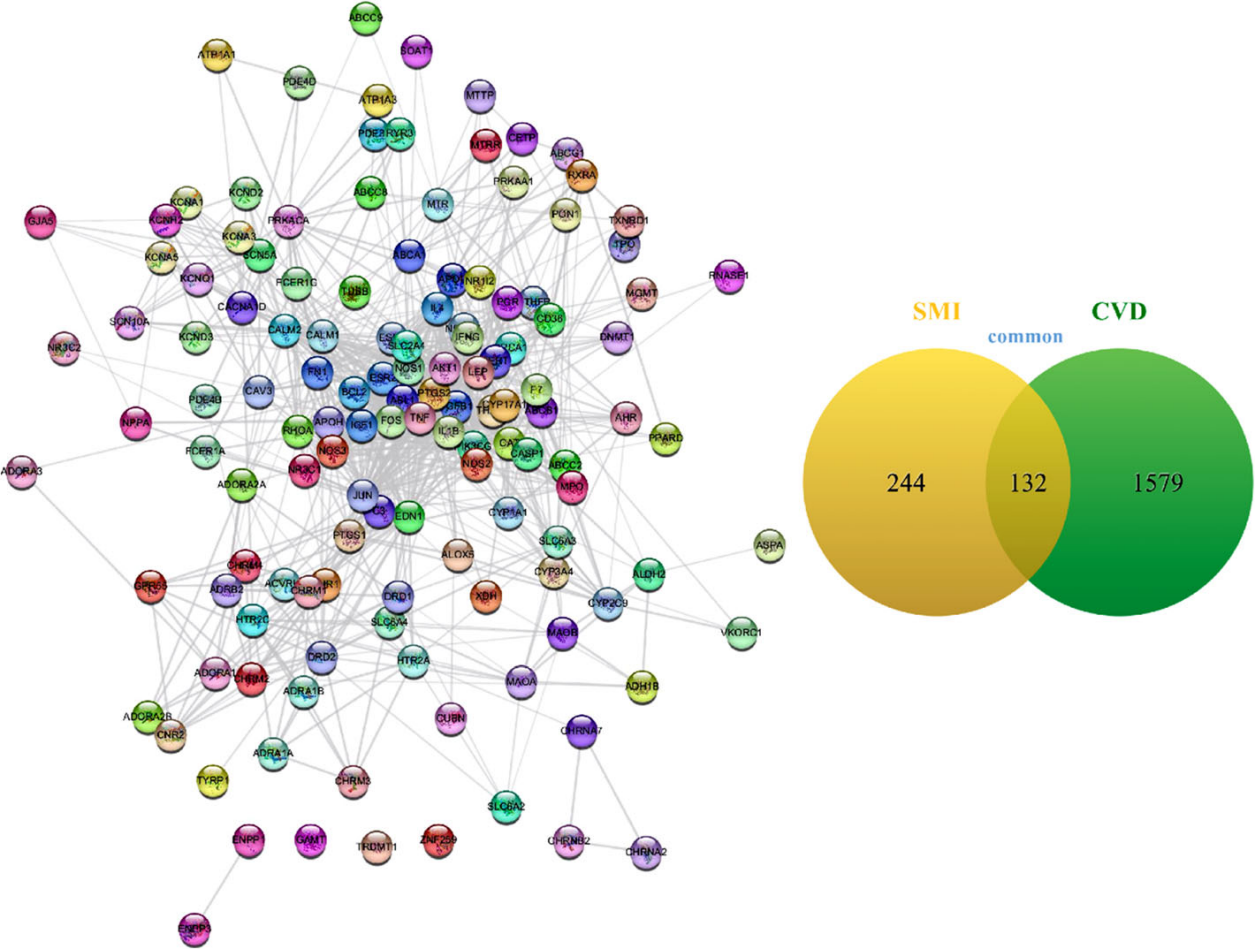
Identification of common targets of CVD and SMI. Common targets of CVD and SMI PPI network. Edges represent protein (node) interactions. Line thickness indicates the strength of data support. Venn diagrams (right) demonstrate the number of shared and unique targets by SMI and CVD

Through mining the TCMSP, BATMAN-TCM, HIT, Drugbank, DisGeNET and OMIM Database, a total of 376 targets related to SMI (Figure S1) and 1711 targets related to CVD (Figure S2) were obtained. Among them, 132 targets were shared by both SMI and CVD (Fig. 2), which were listed in Table 1 (supplementary data), and became the focus of our following analysis.

### Establishment and analysis of Ingredient-Target-Disease & Function-Pathway Network

**Fig. 3:**
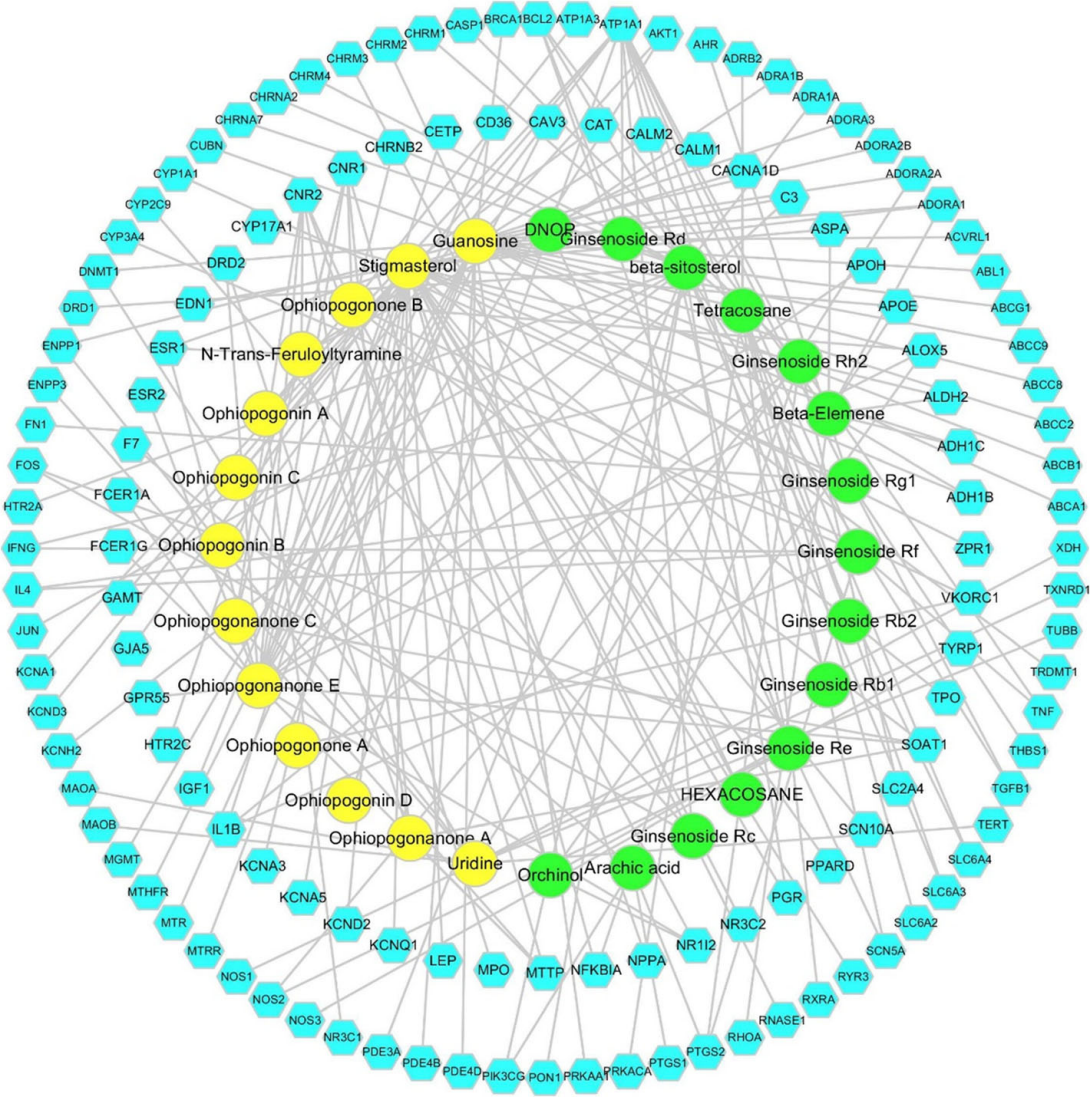
Establishment of ingredient-target network of SMI. Fifteen GR ingredients (green) and thirteen OR ingredients (yellow) cooperatively modulated the 132 common targets (light blue)

**Fig. 4:**
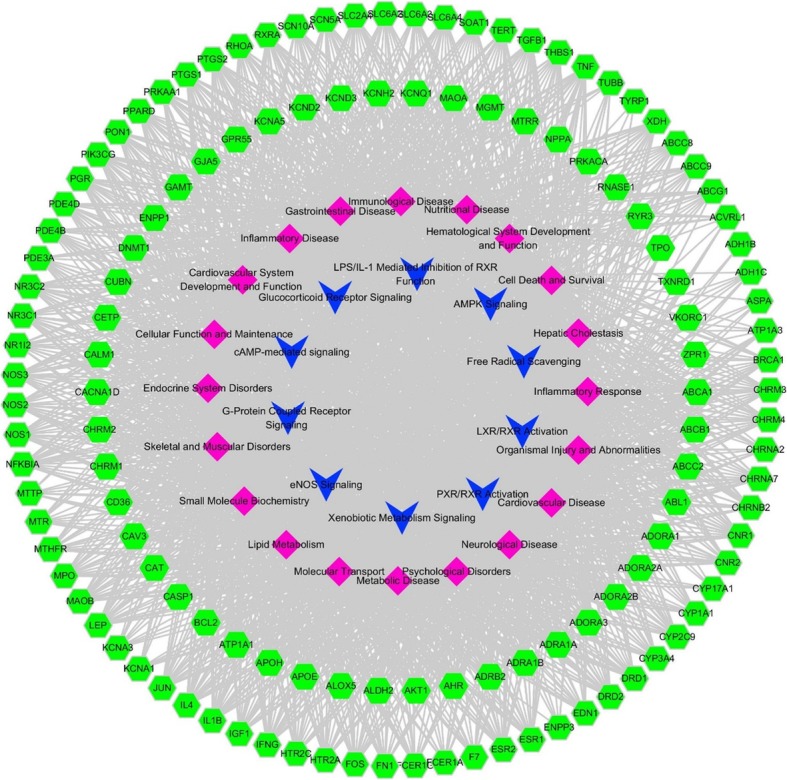
Establishment of target-disease & function-pathway network of SMI. Top 10 canonical pathways (blue), top 20 diseases and bio functions (pink) correlative with the 129 common targets (green) were shown

In order to explain potential pharmacological effects of SMI on the treatment of CVD, compound-target and target-disease & function-pathway networks were performed. SMI ingredient-multiple target network consisted of 160 nodes and 198 edges, which elucidated the 28 ingredients of SMI (15 and 13 derived from GR and OR, respectively) and their modulated 132 common targets. The interactions indicated that one compound could regulate numerous targets, such as beta-sitosterol, ginsenoside Rf, ginsenoside Rg1, ginsenoside Rh2, ginsenoside Rb1, ginsenoside Rd., guanosine, ophiopogonin D, ophiopogonanone E, stigmasterol and so on. In the meanwhile, a single target may be regulated by multiple compounds like ATP1A1, BCL2, CNR1, CNR2, SOAT1, MTTP, AKT1, NOS2, et al. (Fig. 3). Combined with the core analysis results of IPA, a target-disease & function-pathway network was constructed by Cytoscape. This network consisted of 159 nodes (10 pathways, 20 diseases & functions, 129 targets) and 2068 edges, which illustrated the biological process and molecular mechanisms of the common targets (Fig. 4).

**Fig. 5:**
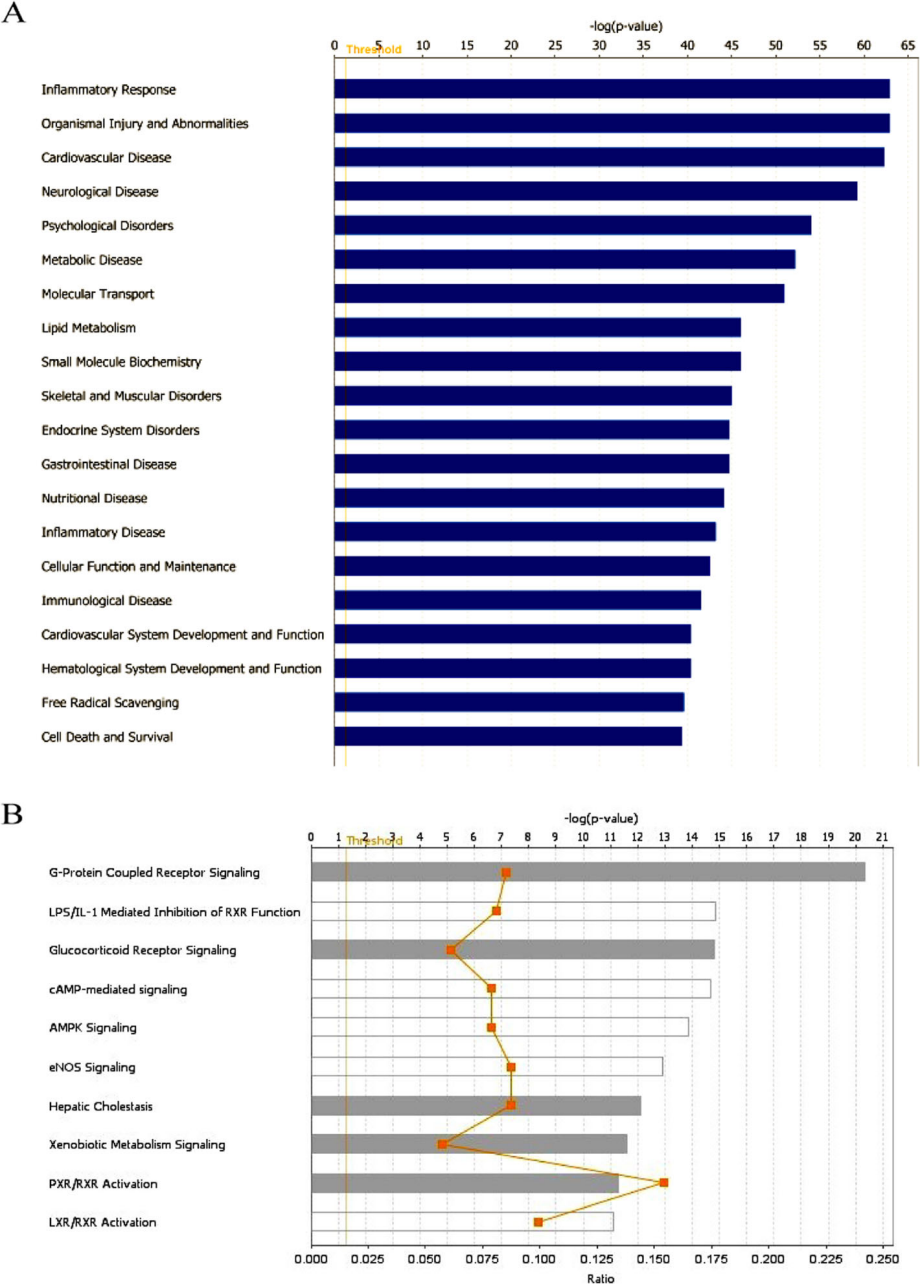
Analysis of target-disease & function-pathway network of SMI. The top 20 diseases & functions (**a**) and the top 10 pathways (**b**) in descending order of -log(*p*-value) score, respectively

**Fig. 6:**
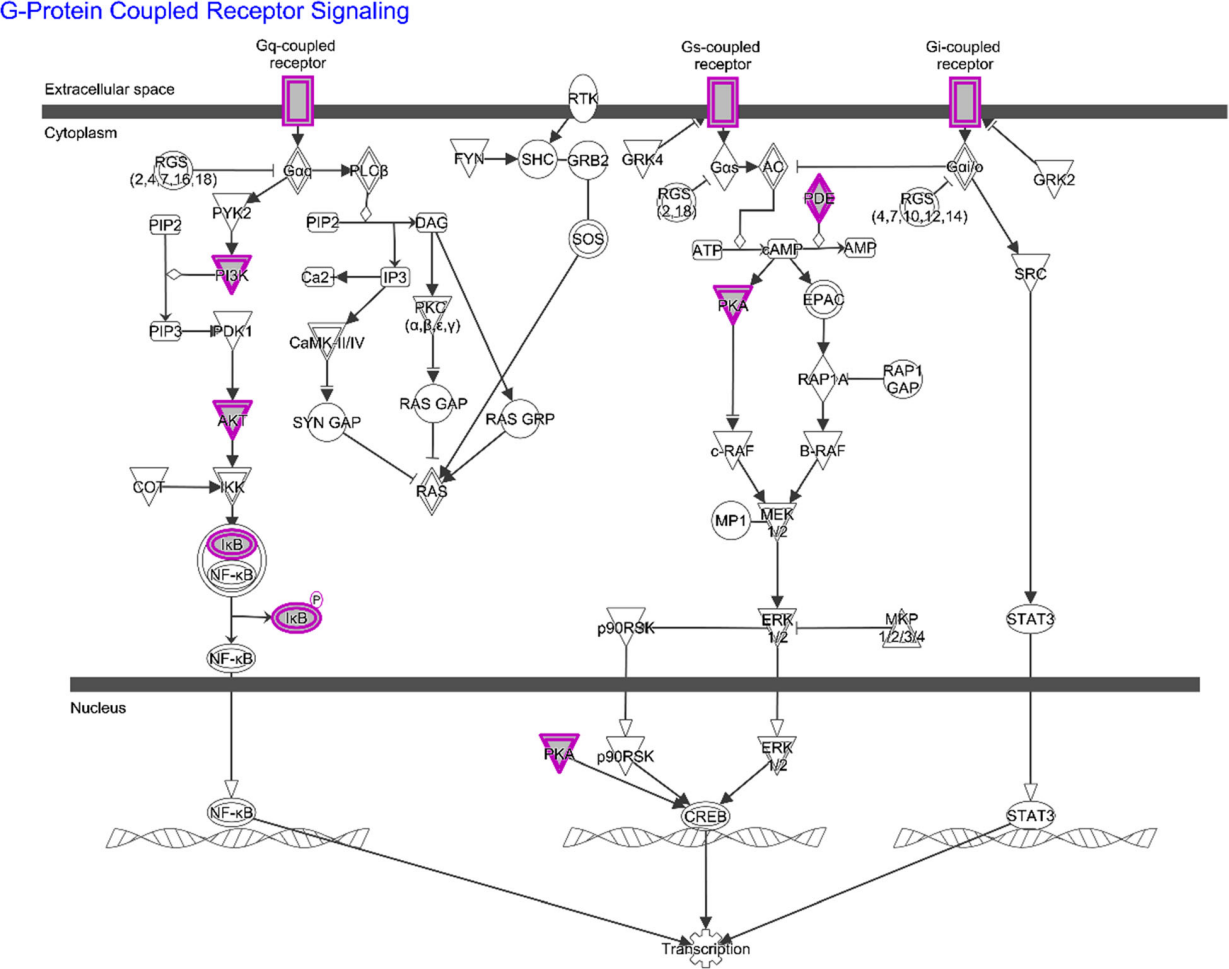
Detailed signaling pathway of G-protein coupled receptor. Targets identified from the 132 common targets were highlighted in purple

The core analysis of diseases & bio functions and canonical pathways were ranked according to *p*-value score. The top 20 diseases & bio functions and top 10 canonical pathways were showed respectively. The top 20 diseases & bio functions by SMI, in descending order of -log (*p*-value) score, were inflammatory response, organismal injury and abnormalities, cardiovascular disease, neurological disease, psychological disorders, metabolic disease, molecular transport, lipid metabolism, small molecule biochemistry, skeletal and muscular disorders, endocrine system disorders, gastrointestinal disease, nutritional disease, inflammatory disease, cellular function and maintenance, immunological disease, cardiovascular system development and function, hematological system development and function, free radical scavenging, cell death and survival. Among them, cardiovascular disease was the third highest one (Fig. 5a). Analysis of IPA canonical pathways showed that the top 10 canonical pathways in descending order of –log (*p*-value) score were G-protein coupled receptor signaling, LPS/IL-1 mediated inhibition of RXR function, glucocorticoid receptor signaling, cAMP-mediated signaling, AMPK signaling, eNOS signaling, hepatic cholestasis, xenobiotic metabolism signaling, PXR/RXR activation and LXR/RXR activation. The G-protein coupled receptor signaling was considered as the essential pathway with the -log (p-value) score of 20.3 (Fig. 5b, Table 2 in supplementary data). The detailed pathways of the G-protein coupled receptor signaling, including the identified molecular targets, were presented in Fig. 6.

**Fig. 7:**
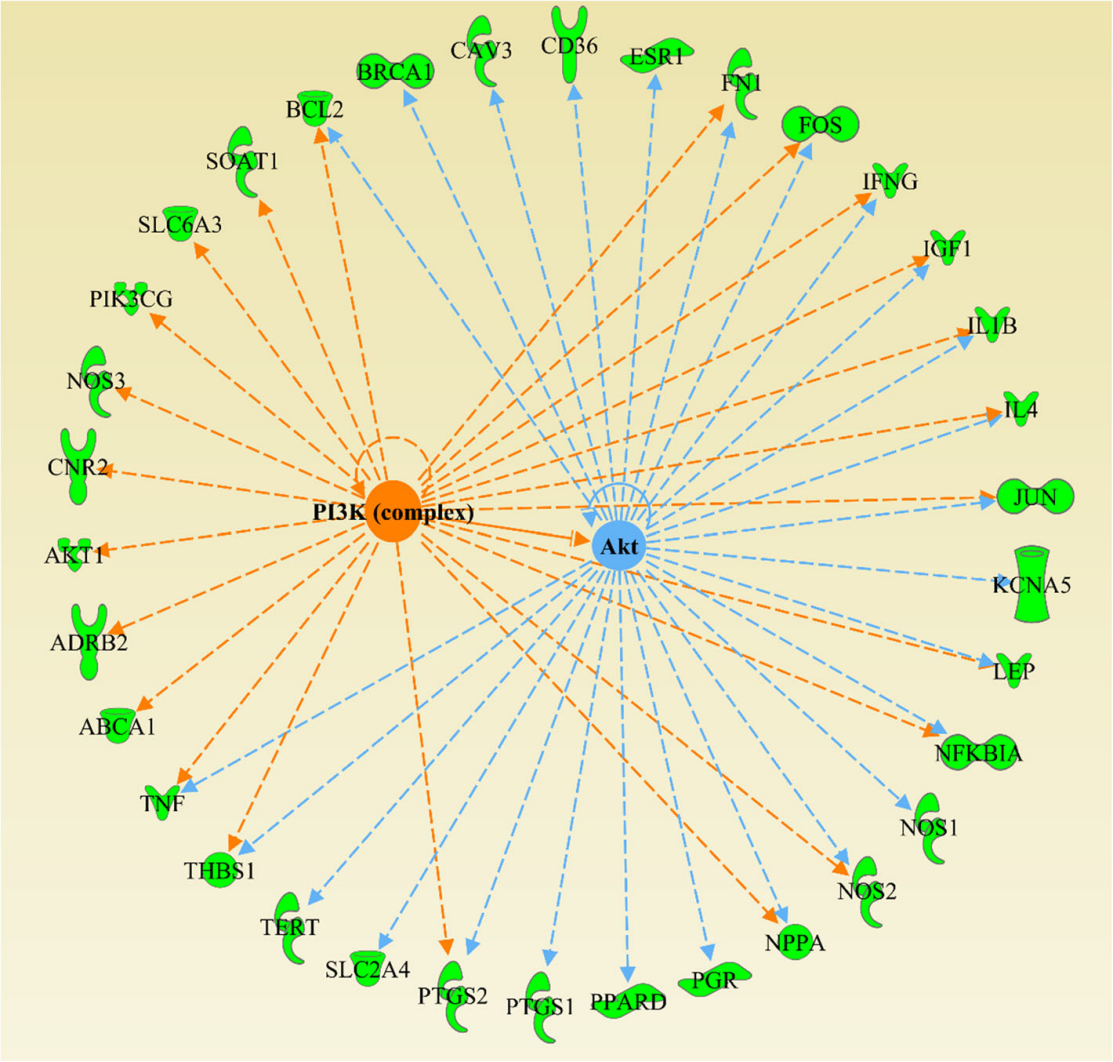
The relationship of PI3K and Akt including their downstream regulated targets

The top 20 upstream regulators and their corresponding targets of the 132 common targets were obtained from “Core analysis” (Table 3 in supplementary data). Among them, PI3K and Akt were shown to have a protein-protein interaction (Fig. 7), which were also the primary targets in the G-protein coupled receptor signaling.

Collectively, the results imply that PI3K-Akt signaling pathway, as the downstream in the G protein-coupled receptor signaling, may serve as CVD-related mechanism for SMI. It has been reported that PI3K/Akt signaling pathway has a direct correlation with DOX-induced cardiotoxicity [29, 30]. Although studies have reported protective effects of SMI on DOX-induced myocardial damage, the underlying molecular mechanisms remain unclear [13–15]. Accordingly, we further explored the cardioprotective mechanism of SMI on DOX-induced cardiomyocyte injury.

### Protective effects of SMI on DOX-induced cell death and apoptosis

**Fig. 8:**
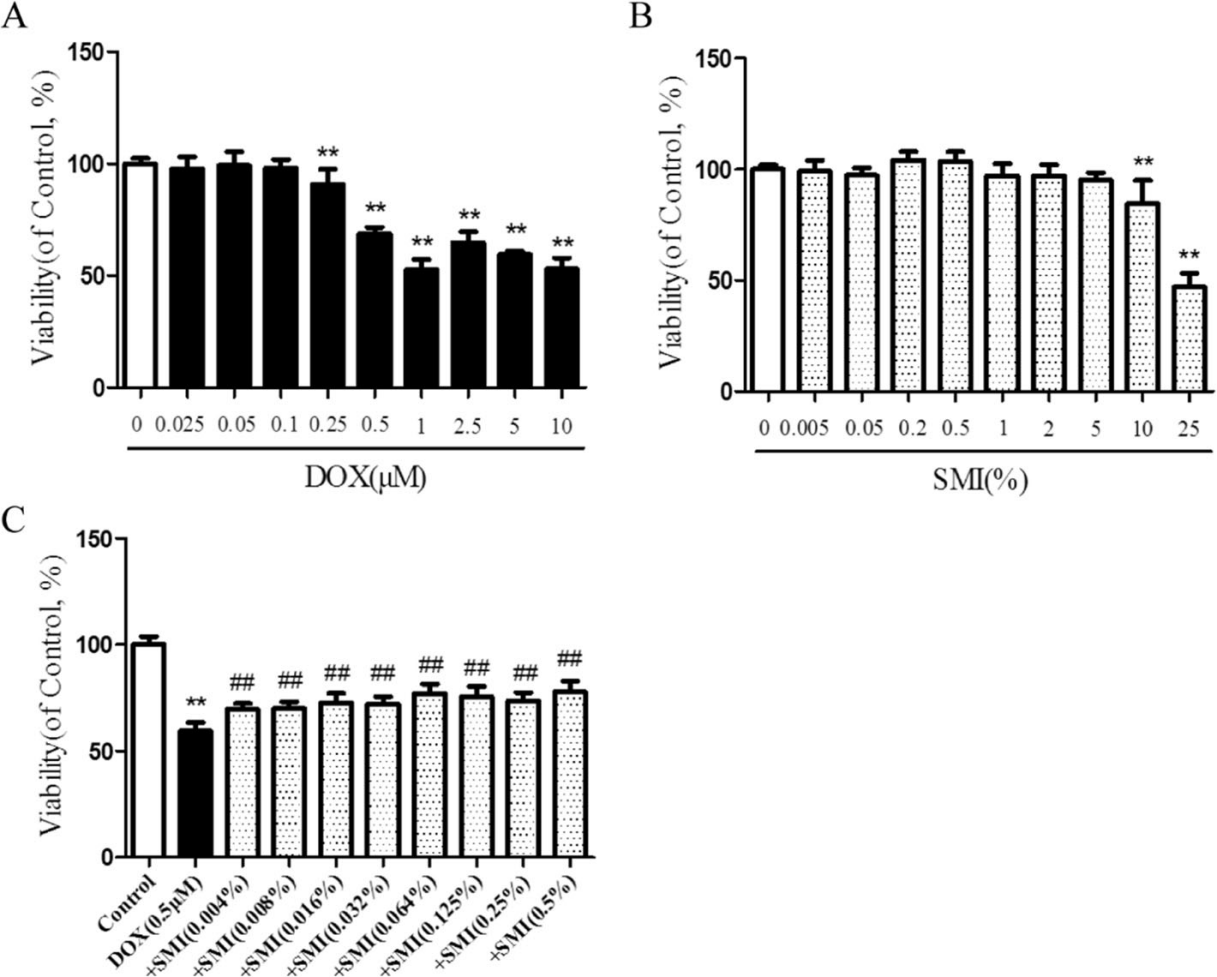
Effects of SMI on DOX-impaired cell viability of H9c2 cells. **a** Toxic effect of DOX on cell viability. **b** Toxic effect of SMI on cell viability. **c** Effects of SMI on the viability of DOX-induced H9c2 cells. **P < 0.01 vs. Control; ^##^*P* < 0.01 vs. DOX

To assess the effect of SMI on DOX-induced cardiotoxicity, we first measured the effects of different concentrations of DOX on the viability of H9c2 cells by CCK8 assay. As shown in Fig. 8a, DOX caused a decrease in the cell viability, when its concentration exceeded 0.25 μM for 24 h (*P* < 0.01). DOX of 0.5–10 μM induced a stable and significant reduction of cell viability. Based on these, a concentration of 0.5 μM was selected and used for subsequent experiment. The cell viability was significantly lower in the high concentration of SMI treatment group (10, 25%) than in the control group (*P* < 0.01); however, no significant difference appeared between the low concentration of SMI (0.005, 0.05, 0.2, 0.5, 1, 2, 5%) and the control group (*P* > 0.05, Fig. 8b). In addition, compared with the cells in DOX (0.5 μM, 24 h) - injured group, treatment with SMI (0.004, 0.008, 0.016, 0.032, 0.064, 0.125, 0.25, 0.5%) significantly increased cell viability (*P* < 0.01, Fig. 8c).

**Fig. 9:**
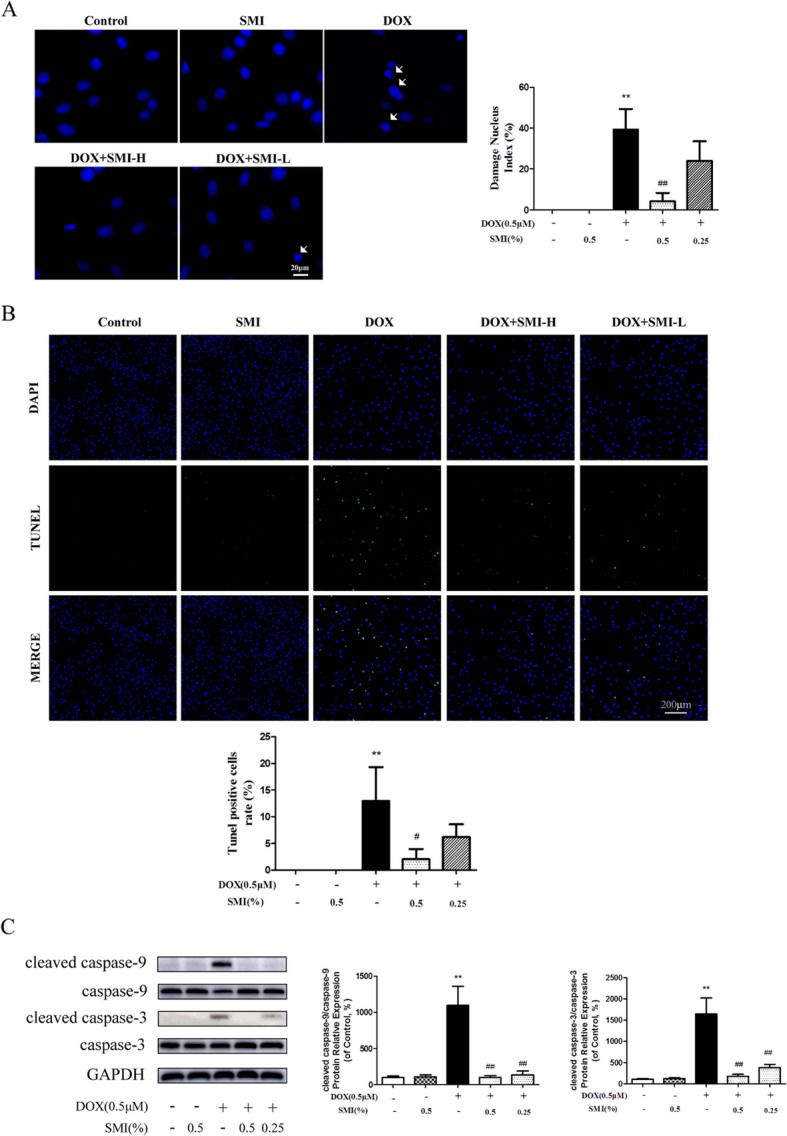
Effects of SMI on DOX-induced apoptosis in H9c2 cells. Cells were pretreated with SMI (0.25, 0.5%) for 24 h, then stimulated with DOX (0.5 μM) for 24 h. **a** Damaged nucleus was examined by Hoechst 33342 staining. Damaged nuclei are marked with white arrows. **b** Cell apoptosis was assessed by TUNEL assay. **c** Protein levels of cleaved caspase-9, caspase-9, cleaved caspase-3 and caspase-3 were measured by Western blot. **P < 0.01 vs. Control; ^#^*P* < 0.05 vs. DOX; ^##^*P* < 0.01 vs. DOX

Hoechst33342 staining assay showed that DOX-induced damaged nuclei, as demonstrated by condensed chromatin, unclear fragments, or apoptosis bodies. However, pre-treatment with SMI (0.5%) significantly decreased the number of damaged nucleus (*P* < 0.01, Fig. 9a). Using TUNEL assay, DNA fragmentation was measured. DOX triggered an increase in TUNEL-positive cells, which were significantly reduced when pretreated with SMI (*P* < 0.01, Fig. 9b).

The activation of caspase-9 and caspase-3 participates in the apoptosis of mitochondrial-dependent pathway. The relative protein levels of cleaved caspase-9 and cleaved caspase-3 were significantly elevated in DOX-treated cells when compared with the control cells (*P* < 0.01, Fig. 9c). However, this effect was significantly inhibited by SMI pretreatment (*P* < 0.01, Fig. 9c).

### Effects of SMI on DOX-induced apoptosis through modulation of the PI3K/Akt signaling pathway

**Fig. 10:**
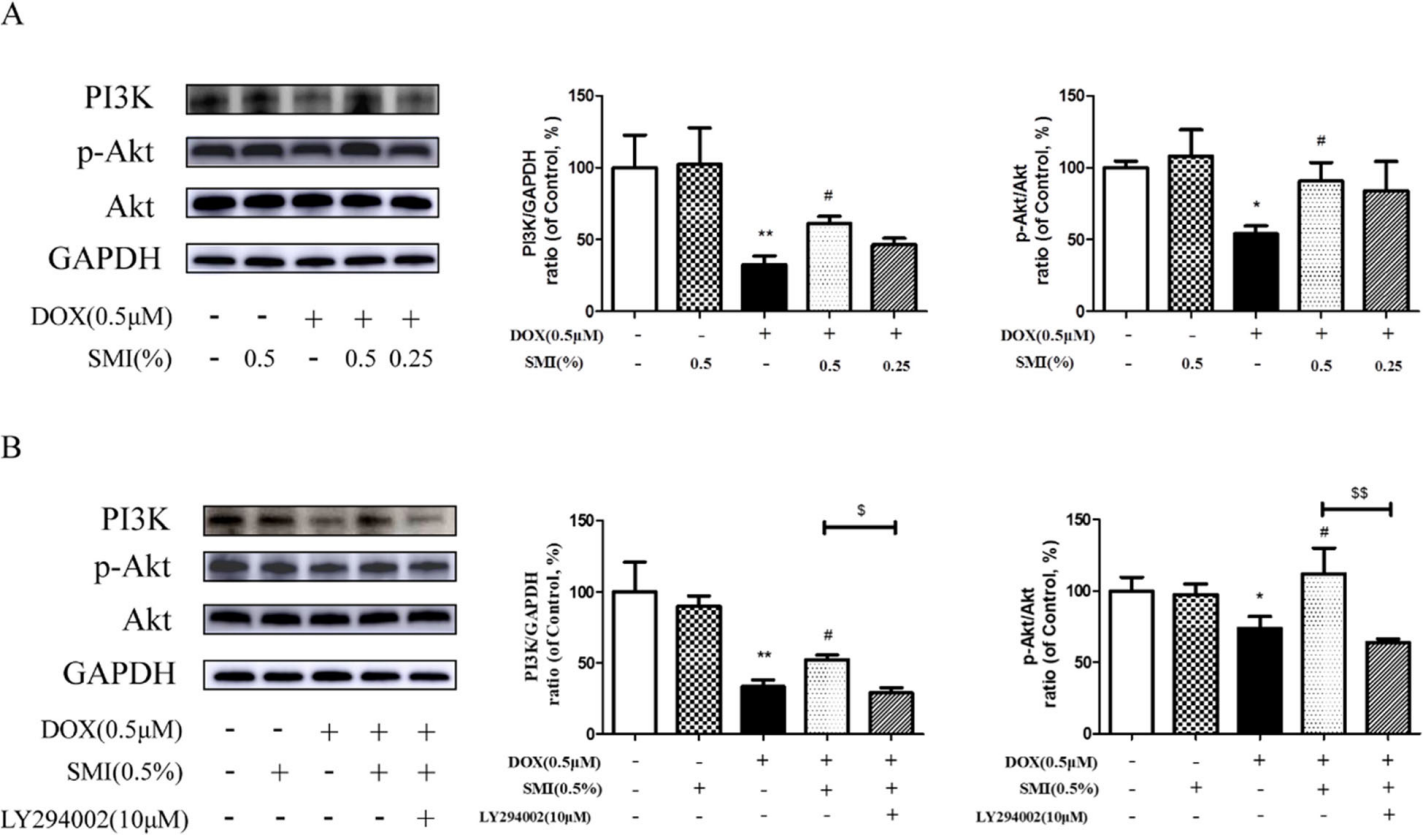
Western blot analysis of PI3K, Akt and p-Akt expression in H9c2 cells. **a** Cells were pretreated with SMI (0.25, 0.5%) for 24 h, then stimulated with DOX (0.5 μM) for 24 h. **b** Cells were pretreated with SMI (0.5%) for 24 h, LY294002 (10 μM) for 1 h, then stimulated with DOX (0.5 μM) for 24 h. PI3K, Akt and p-Akt expression were assessed by Western blot. *P < 0.05 vs. Control; **P < 0.01 vs. Control; ^#^*P* < 0.05 vs. DOX; ^$^*P* < 0.05 DOX + SMI vs. DOX + SMI + LY294002; ^$$^*P* < 0.01 DOX + SMI vs. DOX + SMI + LY294002

To determine the impact of SMI on DOX-induced cardiomyocyte injury, we examined the protein expression of PI3K and p-Akt in H9c2 cells. As depicted in Fig. 10a, the protein levels of PI3K and phosphorylation of Akt were significantly diminished in DOX-treated cells, when compared with the control cells (*P* < 0.01, *P* < 0.05). However, pretreatment of SMI increased the PI3K and p-Akt expression, compared with the DOX group (*P* < 0.05).

**Fig. 11:**
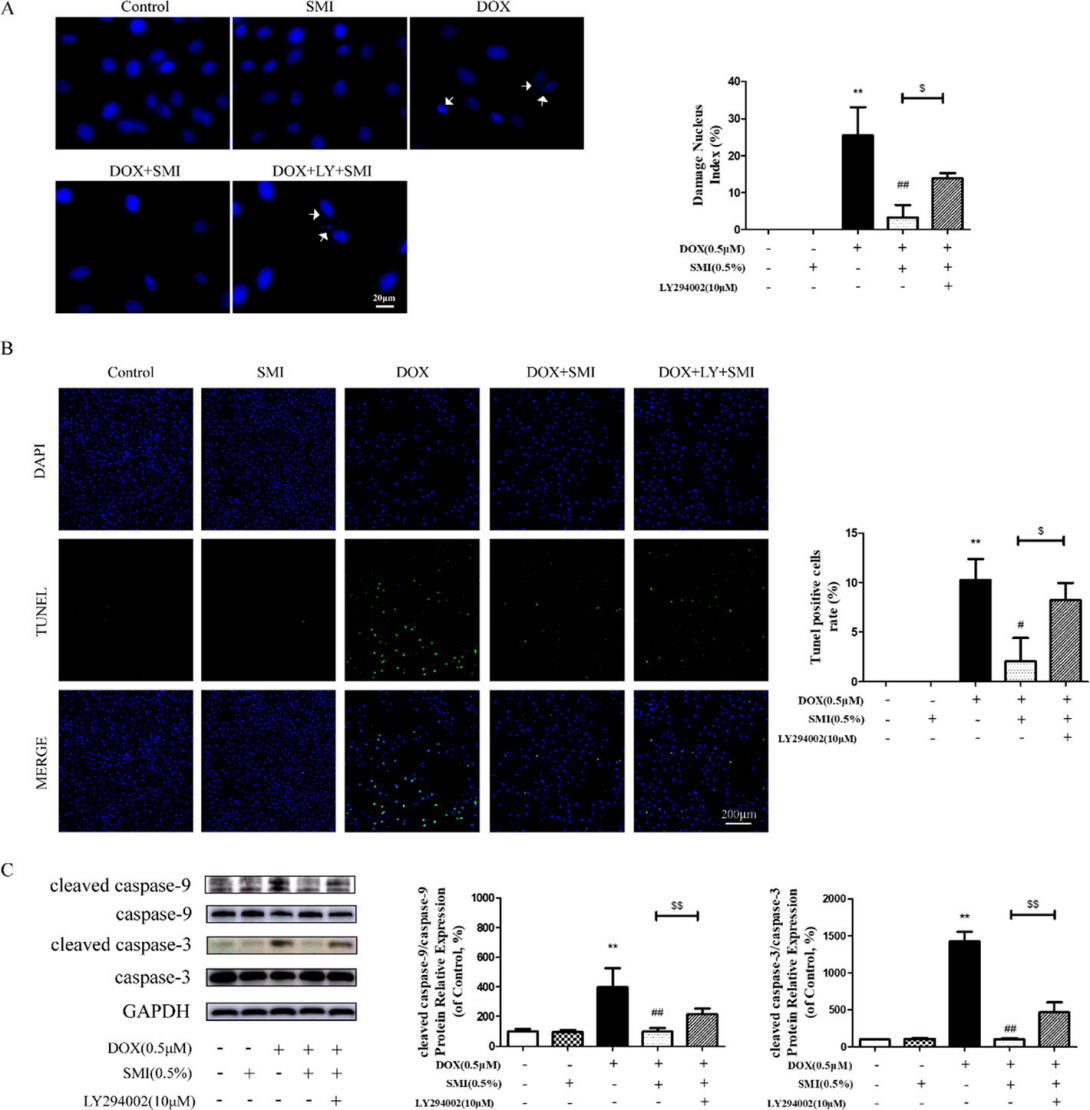
Effects of LY294002 on the protection of SMI on DOX-induced apoptosis in H9c2 cells. Cells were pretreated with SMI (0.5%) for 24 h, LY294002 (10 μM) for 1 h, then stimulated with DOX (0.5 μM) for 24 h. **a** Damaged nucleus was determined by Hoechst 33342 staining. Damaged nuclei are marked with white arrows. **b** Cell apoptosis was determined by TUNEL assay. **c** Protein levels of cleaved caspase-9, caspase-9, cleaved caspase-3 and caspase-3 were assessed by Western blot. **P < 0.01 vs. Control; ^##^*P* < 0.01 vs. DOX; ^$^*P* < 0.05 DOX + SMI vs. DOX + SMI + LY294002; ^$$^*P* < 0.01 DOX + SMI vs. DOX + SMI + LY294002

Moreover, PI3K inhibitor LY294002 blocks the anti-apoptosis effect of SMI, with reduced expression of PI3K and p-Akt (Fig. 10b), increased number of damaged nuclei (Fig. 11a) and apoptosis cells (Fig. 11b) in the SMI pretreatment plus DOX group. As shown in Fig. 11c, DOX dramatically elevated the relative protein levels of cleaved caspase-9 and cleaved caspase-3 (*P* < 0.01), which was markedly suppressed by SMI pretreatment. However, LY294002 significantly inhibited these regulating effects of SMI on caspase-9 and caspase-3 activation (*P* < 0.01, Fig. 11c). These results suggested that PI3K/Akt signaling pathway was involved in SMI-mediated cardiomyocyte protection.

### Protective effects of SMI on DOX-induced Cardiotoxicity in mice

**Fig. 12:**
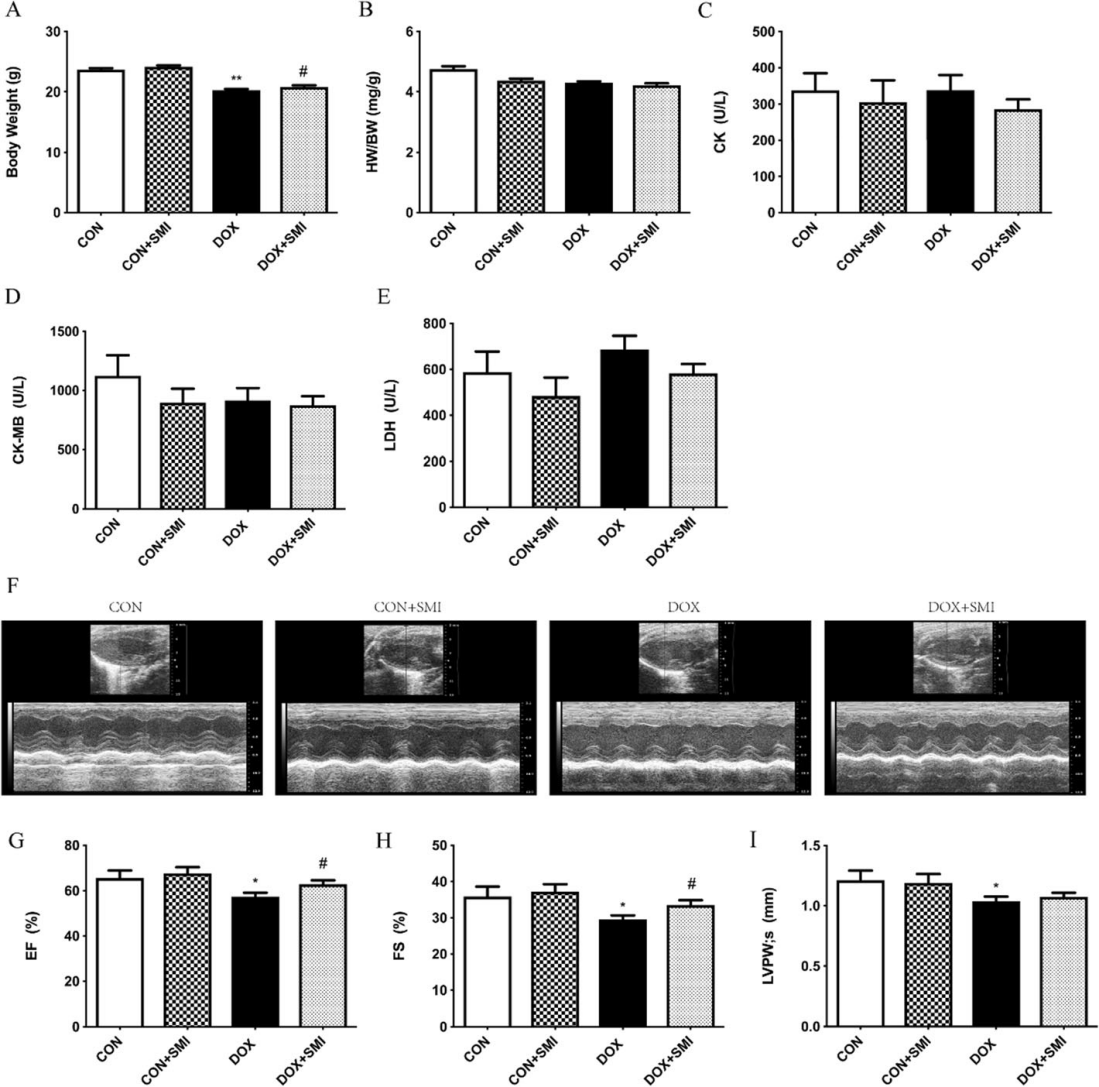
Protective effects of SMI on DOX-induced cardiotoxicity in mice. **a** Body weight. *n* = 10–25. **b** Heart weight to body weight ratios (HW/BW). *n* = 5–15. **c**-**e** The levels of CK, CK-MB and LDH in mouse serum. *n* = 7–22. **f** Representative M-mode echocardiograms of mice. **g**-i Echocardiographic measurement of left ventricular ejection fraction (EF%), fractional shortening (FS%), and left ventricular posterior wall (LVPW; s). *n* = 10–26. *P < 0.05 vs. Control group; **P < 0.01 vs. Control group; ^#^*P* < 0.05 vs. DOX group

We validated the effect of SMI on improvement of cardiac dysfunction in acutely injured mice induced by DOX. SMI pretreatment mitigated the body weight loss induced by DOX (Fig. 12a). The heart-to-body weight ratio (HW/BW) were unchanged among all the experimental mice (Fig. 12b). Although CK, CK-MB and LDH levels were not significantly elevated in DOX-treated mice compared with their levels in normal control mice (Fig. 12c-e), echocardiography assessments showed that levels of EF and FS were significantly decreased in DOX-treated mice, which were markedly inhibited by SMI pretreatment (Fig. 12g, h). Together, these results indicated that SMI improved cardiac dysfunction induced by DOX in mice.

## Discussion

In this study, we showed evidence about the molecular mechanism behind SMI cardioprotective action against DOX-mediated cardiotoxicity using network pharmacology tools. Network pharmacology has been applied in foreseeing the pharmacological mechanisms of TCM [31–33]. Although predictable mechanisms could be obtained from published reports, network pharmacology helps to elucidate the action mechanism of TCM at molecular level with a systematic viewpoint [34]. Therefore, it has been a promising holistic strategy for TCM research. In the present study, network pharmacology study suggested that 28 potential ingredients in SMI may play core roles in regulating the 132 targets, which are major related to CVD. In the target-disease & function-pathway network, the G-protein coupled receptor signaling is an essential pathway. Notably, PI3K and Akt were ranked on the top upstream regulators, which were also the key proteins in G-protein coupled receptor signaling. These suggested that SMI may play a role in the treatment of CVD through the regulation of PI3K/Akt signaling pathway. Even some reports have already revealed that PI3K/Akt pathway played an important role in CVD. The present research of network pharmacology provided a multi-dimensional research strategy for a complicated TCM formula.

PI3K/Akt signaling pathway is critical for multiple biological processes and provides a vital cell survival signal in cardiomyocytes [35]. Downregulation of Akt is associated with DOX-induced damage and apoptosis in cardiomyocytes [36]. PI3K/Akt activation helps to inhibit DOX-induced cardiomyocytes apoptosis. Moreover, it has been reported that Akt activation improved contractile function of failing hearts [37, 38]. Shreds of evidence have shown that SMI and its ingredients ophiopogonin D activated PI3K/Akt signaling pathway in myocardial ischemia-reperfusion injury [39]. Our previous study suggested that ginsenoside Rg3 could promote cell viability and attenuate DOX-induced oxidative damage and apoptosis through Akt activation [40]. Ginsenoside Rb1 presents cardioprotective effect against ischemia/reperfusion injury, which involves activating Akt and phosphorylating glycogen synthase kinase 3β [41].

Several kinds of research have proved the efficacy of SMI on the treatment of DOX cardiotoxicity in the clinic [10, 11]. Although Shengmai injection, composed of the same two components from SMI and schisandra chinensis, has been reported to be reflective of the energy disruption and cardiac dysfunction induced by DOX [42], the underlying mechanism of SMI on DOX-injured myocardium remains to be explored. Dox-induced cardiotoxicity via activation of apoptosis and suppression of survival pathway. The present study dug deeply into the molecular mechanism of SMI treatment on Dox-induced cardiotoxicity. Accordingly, based on network pharmacology prediction, we focused on the PI3K/Akt signaling pathway for the further experiment of cardioprotective effect of SMI on DOX-induced cardiotoxicity.

We utilized H9c2 cells and acutely injured C57BL/6 mice induced by DOX to explore the protective effect of SMI on the myocardium and verify the prediction results of network pharmacology. In vitro, the pretreatment with SMI prevented loss of cell viability, occurance of cell apoptosis, and damage of nucleus, which were induced by DOX. Moreover, the protein level of PI3K and p-Akt was also downregulated by DOX, while pretreatment with SMI increased the protein level of PI3K and p-Akt. To further verify the regulation of PI3K/Akt signaling pathway to the protective effect of SMI, we also utilized PI3K/Akt inhibitor LY294002. This inhibitor disturbed the protective effect of SMI on DOX-induced cardiotoxicity, which demonstrated a reduction in cell viability, an increase in the number of apoptotic cells, and reduced protein level of PI3K and p-Akt. Also, LY294002 markedly suppressed the SMI-mediated activation of caspase-9 and caspase-3, which are involved in mitochondrial-dependent apoptosis pathway. These findings suggest that SMI protects H9c2 cardiomyocytes against DOX-induced cytotoxicity, possibly through the activation of the PI3K/Akt pathway.

Additionally, in vivo, we found that pretreatment of SMI rescued decreased body weight and improved left ventricular function. Of note, in DOX-treated mice, only LDH levels slightly elevated among cardiac enzymes in serum. A possible explanation for this was that after the last administration of DOX, we evaluated left ventricular function, which resulted in the missing of the window of time for CK, CK-MB and LDH appearance [43, 44]. Therefore, the levels of these cardiac enzymes returned to normal. These findings suggest that SMI exhibited markedly cardioprotective effects on DOX-induced acute injury on mice. Future work will be important to examine the therapeutic effect of simultaneous administration of SMI on the chronic toxicity of DOX.

## Conclusions

In conclusion, network pharmacology analysis suggested that the protective effects of SMI may be associated to its 28 potential ingredients, which regulate 132 targets in CVD through modulation of G-protein coupled receptor signaling. In vitro and in vivo results confirmed that SMI attenuated DOX-induced cardiotoxicity and improved DOX-induced cardiac dysfunction. Furthermore, the protective mechanisms possibly involve the activation of the PI3K/Akt signaling pathway. However, further work will be necessary to verify other signaling pathways and elucidate their relationships.

## Supplementary information

**Additional file 1. Supplementary data**.

## Data Availability

We have presented all our main data in the excel sheet in additional file.

## Abbreviations

Akt: Protein kinase B
BATMAN-TCM: Bioinformatics Analysis Tool for Molecular mechANism of Traditional Chinese Medicine
CCK8: Cell counting kit 8
CK: Creatine kinase
CK-MB: Creatine kinase-MB isoenzyme
CVD: Cardiovascular disease
cTnT: Cardiac troponin
DEX: Dexrazoxane
DOX: Doxorubicin
EF: Ejection fraction
FDA: Food and drug administration
FS: Fractional shortening
HIT: Herbal Ingredients’ Targets
HW/BW: Heart-to-body weight ratio
IPA: Ingenuity Pathway Analysis
LDH: Lactate dehydrogenase
LVEF: Left ventricular ejection fraction
OMIM: Online Mendelian Inheritance in Man
PI3K: Phosphoinositide 3-kinase
SMI: Shenmai injection
TCM: Traditional Chinese Medicne
TCMSP: Traditional Chinese Medicine Systems Pharmacology
TUNEL: Terminal deoxynucleotidyl transferase-mediated dUTP nick end labelling
