# Supplementary material for: Investigation of cardiovascular protective effect of Shenmai injection by network pharmacology and pharmacological evaluation
Source: BMC Complement Med Ther. 2020 Apr 15;20:112. doi: 10.1186/s12906-020-02905-8 (PMC7158159; doi:10.1186/s12906-020-02905-8)
Supplement: Supplementary file 1 — Additional file 1. Supplementary data. [file 12906_2020_2905_MOESM1_ESM.docx]

**Table 1. A total of 132 common targets of SMI and CVD.**

| Gene name | Protein name | Uniprot ID |
| --- | --- | --- |
| ABCA1 | ATP-binding cassette sub-family A member 1 | [O95477](https://www.uniprot.org/uniprot/O95477) |
| ABCB1 | Multidrug resistance protein 1 | [P08183](https://www.uniprot.org/uniprot/P08183) |
| ABCC2 | Canalicular multispecific organic anion transporter 1 | [Q92887](https://www.uniprot.org/uniprot/Q92887) |
| ABCC8 | ATP-binding cassette sub-family C member 8 | [Q09428](https://www.uniprot.org/uniprot/Q09428) |
| ABCC9 | ATP-binding cassette sub-family C member 9 | [O60706](https://www.uniprot.org/uniprot/O60706) |
| ABCG1 | ATP-binding cassette sub-family G member 1 | [P45844](https://www.uniprot.org/uniprot/P45844) |
| ABL1 | Tyrosine-protein kinase ABL1 | [P00519](https://www.uniprot.org/uniprot/P00519) |
| ACVRL1 | Serine/threonine-protein kinase receptor R3 | [P37023](https://www.uniprot.org/uniprot/P37023) |
| ADH1B | Alcohol dehydrogenase 1B | [P00325](https://www.uniprot.org/uniprot/P00325) |
| ADH1C | Alcohol dehydrogenase 1C | [P00326](https://www.uniprot.org/uniprot/P00326) |
| ADORA1 | Adenosine receptor A1 | [P30542](https://www.uniprot.org/uniprot/P30542) |
| ADORA2A | Adenosine receptor A2a | [P29274](https://www.uniprot.org/uniprot/P29274) |
| ADORA2B | Adenosine receptor A2b | [P29275](https://www.uniprot.org/uniprot/P29275) |
| ADORA3 | Adenosine receptor A3 | [P0DMS8](https://www.uniprot.org/uniprot/P0DMS8) |
| ADRA1A | Alpha-1A adrenergic receptor | [P35348](https://www.uniprot.org/uniprot/P35348) |
| ADRA1B | Alpha-1B adrenergic receptor | [P35368](https://www.uniprot.org/uniprot/P35368) |
| ADRB2 | Beta-2 adrenergic receptor | [P07550](https://www.uniprot.org/uniprot/P07550) |
| AHR | Aryl hydrocarbon receptor | [P35869](https://www.uniprot.org/uniprot/P35869) |
| AKT1 | RAC-alpha serine/threonine-protein kinase | [P31749](https://www.uniprot.org/uniprot/P31749) |
| ALDH2 | Aldehyde dehydrogenase, mitochondrial | [P05091](https://www.uniprot.org/uniprot/P05091) |
| ALOX5 | Arachidonate 5-lipoxygenase | [P09917](https://www.uniprot.org/uniprot/P09917) |
| APOE | Apolipoprotein E | [P02649](https://www.uniprot.org/uniprot/P02649) |
| APOH | Beta-2-glycoprotein 1 | [P02749](https://www.uniprot.org/uniprot/P02749) |
| ASPA | Aspartoacylase | [P45381](https://www.uniprot.org/uniprot/P45381) |
| ATP1A1 | Sodium/potassium-transporting ATPase subunit alpha-1 | [P05023](https://www.uniprot.org/uniprot/P05023) |
| ATP1A3 | Sodium/potassium-transporting ATPase subunit alpha-3 | [P13637](https://www.uniprot.org/uniprot/P13637) |
| BCL2 | Apoptosis regulator Bcl-2 | [P10415](https://www.uniprot.org/uniprot/P10415) |
| BRCA1 | Breast cancer type 1 susceptibility protein | [P38398](https://www.uniprot.org/uniprot/P38398) |
| C3 | Complement C3 | [P01024](https://www.uniprot.org/uniprot/P01024) |
| CACNA1D | Voltage-dependent L-type calcium channel subunit alpha-1D | [Q01668](https://www.uniprot.org/uniprot/Q01668) |
| CALM1 | Calmodulin-1 | [P0DP23](https://www.uniprot.org/uniprot/P0DP23) |
| CALM2 | Calmodulin-2 | [P0DP24](https://www.uniprot.org/uniprot/P0DP24) |
| CASP1 | Caspase-1 | [P29466](https://www.uniprot.org/uniprot/P29466) |
| CAT | Catalase | [P04040](https://www.uniprot.org/uniprot/P04040) |
| CAV3 | Caveolin-3 | [P56539](https://www.uniprot.org/uniprot/P56539) |
| CD36 | Platelet glycoprotein 4 | [P16671](https://www.uniprot.org/uniprot/P16671) |
| CETP | Cholesteryl ester transfer protein | [P11597](https://www.uniprot.org/uniprot/P11597) |
| CHRM1 | Muscarinic acetylcholine receptor M1 | [P11229](https://www.uniprot.org/uniprot/P11229) |
| CHRM2 | Muscarinic acetylcholine receptor M2 | [P08172](https://www.uniprot.org/uniprot/P08172) |
| CHRM3 | Muscarinic acetylcholine receptor M3 | [P20309](https://www.uniprot.org/uniprot/P20309) |
| CHRM4 | Muscarinic acetylcholine receptor M4 | [P08173](https://www.uniprot.org/uniprot/P08173) |
| CHRNA2 | Neuronal acetylcholine receptor subunit alpha-2 | [Q15822](https://www.uniprot.org/uniprot/Q15822) |
| CHRNA7 | Neuronal acetylcholine receptor subunit alpha-7 | [P36544](https://www.uniprot.org/uniprot/P36544) |
| CHRNB2 | Neuronal acetylcholine receptor subunit beta-2 | [P17787](https://www.uniprot.org/uniprot/P17787) |
| CNR1 | Cannabinoid receptor 1 | [P21554](https://www.uniprot.org/uniprot/P21554) |
| CNR2 | Cannabinoid receptor 2 | [P34972](https://www.uniprot.org/uniprot/P34972) |
| CUBN | Cubilin | [O60494](https://www.uniprot.org/uniprot/O60494) |
| CYP17A1 | Steroid 17-alpha-hydroxylase/17,20 lyase | [P05093](https://www.uniprot.org/uniprot/P05093) |
| CYP1A1 | Cytochrome P450 1A1 | [P04798](https://www.uniprot.org/uniprot/P04798) |
| CYP2C9 | Cytochrome P450 2C9 | [P11712](https://www.uniprot.org/uniprot/P11712) |
| CYP3A4 | Cytochrome P450 3A4 | [P08684](https://www.uniprot.org/uniprot/P08684) |
| DNMT1 | DNA (cytosine-5)-methyltransferase 1 | [P26358](https://www.uniprot.org/uniprot/P26358) |
| DRD1 | D(1A) dopamine receptor | [P21728](https://www.uniprot.org/uniprot/P21728) |
| DRD2 | D(2) dopamine receptor | [P14416](https://www.uniprot.org/uniprot/P14416) |
| EDN1 | Endothelin-1 | [P05305](https://www.uniprot.org/uniprot/P05305) |
| ENPP1 | Ectonucleotide pyrophosphatase/phosphodiesterase family member 1 | [P22413](https://www.uniprot.org/uniprot/P22413) |
| ENPP3 | Ectonucleotide pyrophosphatase/phosphodiesterase family member 3 | [O14638](https://www.uniprot.org/uniprot/O14638) |
| ESR1 | Estrogen receptor | [P03372](https://www.uniprot.org/uniprot/P03372) |
| ESR2 | Estrogen receptor beta | [Q92731](https://www.uniprot.org/uniprot/Q92731) |
| F7 | Coagulation factor VII | [P08709](https://www.uniprot.org/uniprot/P08709) |
| FCER1A | High affinity immunoglobulin epsilon receptor subunit alpha | [P12319](https://www.uniprot.org/uniprot/P12319) |
| FCER1G | High affinity immunoglobulin epsilon receptor subunit gamma | [P30273](https://www.uniprot.org/uniprot/P30273) |
| FN1 | Fibronectin | [P02751](https://www.uniprot.org/uniprot/P02751) |
| FOS | Proto-oncogene c-Fos | [P01100](https://www.uniprot.org/uniprot/P01100) |
| GAMT | Guanidinoacetate N-methyltransferase | [Q14353](https://www.uniprot.org/uniprot/Q14353) |
| GJA5 | Gap junction alpha-5 protein | [P36382](https://www.uniprot.org/uniprot/P36382) |
| GPR55 | G-protein coupled receptor 55 | [Q9Y2T6](https://www.uniprot.org/uniprot/Q9Y2T6) |
| HTR2A | 5-hydroxytryptamine receptor 2A | [P28223](https://www.uniprot.org/uniprot/P28223) |
| HTR2C | 5-hydroxytryptamine receptor 2C | [P28335](https://www.uniprot.org/uniprot/P28335) |
| IFNG | Interferon gamma | [P01579](https://www.uniprot.org/uniprot/P01579) |
| IGF1 | Insulin-like growth factor I | [P05019](https://www.uniprot.org/uniprot/P05019) |
| IL1B | Interleukin-1 beta | [P01584](https://www.uniprot.org/uniprot/P01584) |
| IL4 | Interleukin-4 | [P05112](https://www.uniprot.org/uniprot/P05112) |
| JUN | Transcription factor AP-1 | [P05412](https://www.uniprot.org/uniprot/P05412) |
| KCNA1 | Potassium voltage-gated channel subfamily A member 1 | [Q09470](https://www.uniprot.org/uniprot/Q09470) |
| KCNA3 | Potassium voltage-gated channel subfamily A member 3 | [P22001](https://www.uniprot.org/uniprot/P22001) |
| KCNA5 | Potassium voltage-gated channel subfamily A member 5 | [P22460](https://www.uniprot.org/uniprot/P22460) |
| KCND2 | Potassium voltage-gated channel subfamily D member 2 | [Q9NZV8](https://www.uniprot.org/uniprot/Q9NZV8) |
| KCND3 | Potassium voltage-gated channel subfamily D member 3 | [Q9UK17](https://www.uniprot.org/uniprot/Q9UK17) |
| KCNH2 | Potassium voltage-gated channel subfamily H member 2 | [Q12809](https://www.uniprot.org/uniprot/Q12809) |
| KCNQ1 | Potassium voltage-gated channel subfamily KQT member 1 | [P51787](https://www.uniprot.org/uniprot/P51787) |
| LEP | Leptin | [P41159](https://www.uniprot.org/uniprot/P41159) |
| MAOA | Amine oxidase [flavin-containing] A | [P21397](https://www.uniprot.org/uniprot/P21397) |
| MAOB | Amine oxidase [flavin-containing] B | [P27338](https://www.uniprot.org/uniprot/P27338) |
| MGMT | Methylated-DNA--protein-cysteine methyltransferase | [P16455](https://www.uniprot.org/uniprot/P16455) |
| MPO | Myeloperoxidase | [P05164](https://www.uniprot.org/uniprot/P05164) |
| MTHFR | Methylenetetrahydrofolate reductase | [P42898](https://www.uniprot.org/uniprot/P42898) |
| MTR | Methionine synthase | [Q99707](https://www.uniprot.org/uniprot/Q99707) |
| MTRR | Methionine synthase reductase | [Q9UBK8](https://www.uniprot.org/uniprot/Q9UBK8) |
| MTTP | Microsomal triglyceride transfer protein large subunit | [P55157](https://www.uniprot.org/uniprot/P55157) |
| NFKBIA | NF-kappa-B inhibitor alpha | [P25963](https://www.uniprot.org/uniprot/P25963) |
| NOS1 | Nitric oxide synthase, brain | [P29475](https://www.uniprot.org/uniprot/P29475) |
| NOS2 | Nitric oxide synthase, inducible | [P35228](https://www.uniprot.org/uniprot/P35228) |
| NOS3 | Nitric oxide synthase, endothelial | [P29474](https://www.uniprot.org/uniprot/P29474) |
| NPPA | Natriuretic peptides A | [P01160](https://www.uniprot.org/uniprot/P01160) |
| NR1I2 | Nuclear receptor subfamily 1 group I member 2 | [O75469](https://www.uniprot.org/uniprot/O75469) |
| NR3C1 | Glucocorticoid receptor | [P04150](https://www.uniprot.org/uniprot/P04150) |
| NR3C2 | Mineralocorticoid receptor | [P08235](https://www.uniprot.org/uniprot/P08235) |
| PDE3A | cGMP-inhibited 3',5'-cyclic phosphodiesterase A | [Q14432](https://www.uniprot.org/uniprot/Q14432) |
| PDE4B | cAMP-specific 3',5'-cyclic phosphodiesterase 4B | [Q07343](https://www.uniprot.org/uniprot/Q07343) |
| PDE4D | cAMP-specific 3',5'-cyclic phosphodiesterase 4D | [Q08499](https://www.uniprot.org/uniprot/Q08499) |
| PGR | Progesterone receptor | [P06401](https://www.uniprot.org/uniprot/P06401) |
| PIK3CG | Phosphatidylinositol 4,5-bisphosphate 3-kinase catalytic subunit gamma isoform | [P48736](https://www.uniprot.org/uniprot/P48736) |
| PON1 | Serum paraoxonase/arylesterase 1 | [P27169](https://www.uniprot.org/uniprot/P27169) |
| PPARD | Peroxisome proliferator-activated receptor delta | [Q03181](https://www.uniprot.org/uniprot/Q03181) |
| PRKAA1 | 5'-AMP-activated protein kinase catalytic subunit alpha-1 | [Q13131](https://www.uniprot.org/uniprot/Q13131) |
| PRKACA | cAMP-dependent protein kinase catalytic subunit alpha | [P17612](https://www.uniprot.org/uniprot/P17612) |
| PTGS1 | Prostaglandin G/H synthase 1 | [P23219](https://www.uniprot.org/uniprot/P23219) |
| PTGS2 | Prostaglandin G/H synthase 2 | [P35354](https://www.uniprot.org/uniprot/P35354) |
| RHOA | Transforming protein RhoA | [P61586](https://www.uniprot.org/uniprot/P61586) |
| RNASE1 | Ribonuclease pancreatic | [P07998](https://www.uniprot.org/uniprot/P07998) |
| RXRA | Retinoic acid receptor RXR-alpha | [P19793](https://www.uniprot.org/uniprot/P19793) |
| RYR3 | Ryanodine receptor 3 | [Q15413](https://www.uniprot.org/uniprot/Q15413) |
| SCN10A | Sodium channel protein type 10 subunit alpha | [Q9Y5Y9](https://www.uniprot.org/uniprot/Q9Y5Y9) |
| SCN5A | Sodium channel protein type 5 subunit alpha | [Q14524](https://www.uniprot.org/uniprot/Q14524) |
| SLC2A4 | Solute carrier family 2, facilitated glucose transporter member 4 | [P14672](https://www.uniprot.org/uniprot/P14672) |
| SLC6A2 | Sodium-dependent noradrenaline transporter | [P23975](https://www.uniprot.org/uniprot/P23975) |
| SLC6A3 | Sodium-dependent dopamine transporter | [Q01959](https://www.uniprot.org/uniprot/Q01959) |
| SLC6A4 | Sodium-dependent serotonin transporter | [P31645](https://www.uniprot.org/uniprot/P31645) |
| SOAT1 | Sterol O-acyltransferase 1 | [P35610](https://www.uniprot.org/uniprot/P35610) |
| TERT | Telomerase reverse transcriptase | [O14746](https://www.uniprot.org/uniprot/O14746) |
| TGFB1 | Transforming growth factor beta-1 proprotein | [P01137](https://www.uniprot.org/uniprot/P01137) |
| THBS1 | Thrombospondin-1 | [P07996](https://www.uniprot.org/uniprot/P07996) |
| TNF | Tumor necrosis factor | [P01375](https://www.uniprot.org/uniprot/P01375) |
| TPO | Thyroid peroxidase | [P07202](https://www.uniprot.org/uniprot/P07202) |
| TRDMT1 | tRNA (cytosine(38)-C(5))-methyltransferase | [O14717](https://www.uniprot.org/uniprot/O14717) |
| TUBB | Tubulin beta chain | [P07437](https://www.uniprot.org/uniprot/P07437) |
| TXNRD1 | Thioredoxin reductase 1, cytoplasmic | [Q16881](https://www.uniprot.org/uniprot/Q16881) |
| TYRP1 | 5,6-dihydroxyindole-2-carboxylic acid oxidase | [P17643](https://www.uniprot.org/uniprot/P17643) |
| VKORC1 | Vitamin K epoxide reductase complex subunit 1 | [Q9BQB6](https://www.uniprot.org/uniprot/Q9BQB6) |
| XDH | Xanthine dehydrogenase/oxidase | [P47989](https://www.uniprot.org/uniprot/P47989) |
| ZPR1 | Zinc finger protein ZPR1 | [O75312](https://www.uniprot.org/uniprot/O75312) |

**Table 2. Top 10 canonical pathways and their correlative targets.**

| Ingenuity Canonical Pathways | -log(p-value) | Ratio | Molecules |
| --- | --- | --- | --- |
| G-Protein Coupled Receptor Signaling | 20.30 | 8.51E-02 | PDE3A,CNR1,CHRM4,ADORA3,PDE4B,DRD2,CHRM1,CHRM3,HTR2C,CHRM2,NFKBIA,AKT1,DRD1,CNR2,PIK3CG,PRKACA,ADORA2B,PDE4D,ADORA1,ADORA2A,ADRA1B,ADRA1A,ADRB2,HTR2A |
| LPS/IL-1 Mediated Inhibition of RXR Function | 14.90 | 8.07E-02 | APOE,ABCB1,MGMT,ABCC2,CYP2C9,ABCG1,ABCA1,CETP,ALDH2,MAOB,JUN,CYP3A4,NR1I2,CAT,IL1B,RXRA,TNF,MAOA |
| Glucocorticoid Receptor Signaling | 14.80 | 6.09E-02 | IFNG,NR3C1,BCL2,PGR,FOS,NFKBIA,AKT1,JUN,TGFB1,PIK3CG,PRKACA,PRKAA1,IL1B,NR3C2,PTGS2,NPPA,NOS2,TNF,ESR1,ADRB2,IL4 |
| cAMP-mediated signaling | 14.70 | 7.89E-02 | PDE3A,CNR1,CHRM4,ADORA3,PDE4B,DRD2,CHRM1,CHRM3,CHRM2,CALM1 (includes others),DRD1,CNR2,PRKACA,ADORA2B,PDE4D,ADORA1,ADORA2A,ADRB2 |
| AMPK Signaling | 13.90 | 7.87E-02 | LEP,CHRM4,NOS3,SLC2A4,CHRM1,CHRM3,CHRM2,AKT1,CHRNB2,PIK3CG,PRKACA,PRKAA1,CHRNA7,CHRNA2,ADRA1B,ADRA1A,ADRB2 |
| eNOS Signaling | 12.90 | 8.72E-02 | CHRM4,NOS3,CHRM3,CHRM1,CALM1 (includes others),CHRM2,AKT1,CHRNB2,PIK3CG,PRKAA1,PRKACA,CHRNA7,CHRNA2,ESR2,ESR1 |
| Hepatic Cholestasis | 12.10 | 8.75E-02 | ABCB1,IFNG,ABCC2,CETP,JUN,NFKBIA,NR1I2,TGFB1,PRKACA,IL1B,RXRA,TNF,ESR1,IL4 |
| Xenobiotic Metabolism Signaling | 11.60 | 5.72E-02 | ABCB1,CYP1A1,MGMT,ABCC2,CYP2C9,ALDH2,MAOB,CYP3A4,NR1I2,PIK3CG,CAT,IL1B,NOS2,RXRA,TNF,AHR,MAOA |
| PXR/RXR Activation | 11.30 | 1.54E-01 | ABCB1,AKT1,CYP3A4,NR1I2,ABCC2,PRKACA,CYP2C9,RXRA,NR3C1,TNF |
| LXR/RXR Activation | 11.10 | 9.92E-02 | PON1,APOE,APOH,CD36,IL1B,ABCG1,PTGS2,NOS2,RXRA,TNF,ABCA1,CETP |

**Table 3.** **Top 20 upstream regulators and their downstream regulated targets.**

| No. | Upstream Regulator | Molecule Type | p-value of overlap | Target molecules in dataset | Mechanistic Network |
| --- | --- | --- | --- | --- | --- |
| 1 | SP1 | transcription regulator | 4.40E-30 | ABCA1,ABCB1,ABCC8,ACVRL1,ADRA1B,ALOX5,APOE,BCL2,CAT,CETP,CYP17A1,DRD1,DRD2,ESR1,F7,FN1,FOS,IFNG,IGF1,IL1B,JUN,KCNA3,MAOA,MAOB,NFKBIA,NOS1,NOS3,NPPA,PGR,PPARD,PTGS1,PTGS2,SLC6A2,SOAT1,TERT,TGFB1,TNF,TXNRD1 | 82 (19) |
| 2 | TNF | cytokine | 7.73E-30 | ABCA1,ABCC2,ADORA1,ADORA2A,ADORA2B,ADRB2,ALDH2,ALOX5,APOE,ATP1A1,BCL2,CASP1,CAT,CD36,CNR2,CYP17A1,CYP1A1,DNMT1,EDN1,ENPP3,ESR1,FCER1G,FN1,FOS,IFNG,IGF1,IL1B,IL4,JUN,KCNH2,LEP,MGMT,MPO,MTTP,NFKBIA,NOS1,NOS2,NOS3,NPPA,NR1I2,NR3C1,PDE4B,PIK3CG,PPARD,PTGS1,PTGS2,RHOA,RNASE1,RXRA,SLC2A4,SOAT1,TERT,TGFB1,THBS1,TNF,TPO,TXNRD1,XDH | 93 (18) |
| 3 | TGFB1 | growth factor | 4.25E-24 | ABCA1,ABCC2,ABCG1,ABL1,ACVRL1,ADORA1,ADORA2B,AHR,AKT1,ALDH2,ALOX5,APOE,BCL2,CALM1 (includes others),CASP1,CAT,CD36,CHRNA7,CYP17A1,DNMT1,EDN1,ENPP1,ESR2,FCER1A,FCER1G,FN1,FOS,IFNG,IGF1,IL1B,IL4,JUN,LEP,MAOA,MGMT,MTRR,NFKBIA,NOS2,NOS3,NPPA,PDE4D,PPARD,PTGS1,PTGS2,RHOA,RXRA,TERT,TGFB1,THBS1,TNF,TXNRD1,XDH | 92 (21) |
| 4 | Insulin | group | 4.69E-24 | AKT1,APOE,BCL2,CD36,CHRM1,CHRM3,CYP17A1,CYP1A1,CYP2C9,EDN1,FN1,FOS,IGF1,JUN,LEP,MAOA,MAOB,MTTP,NFKBIA,NOS2,NOS3,NR3C1,PPARD,SLC2A4,SLC6A3,SLC6A4,TGFB1,TNF,TPO | 85 (21) |
| 5 | Akt | group | 6.43E-24 | BCL2,BRCA1,CAV3,CD36,ESR1,FN1,FOS,IFNG,IGF1,IL1B,IL4,JUN,KCNA5,LEP,NFKBIA,NOS1,NOS2,NPPA,PGR,PPARD,PTGS1,PTGS2,SLC2A4,TERT,THBS1,TNF | 79 (21) |
| 6 | IL1B | cytokine | 7.17E-24 | ABCC2,ADORA2B,ADRB2,APOE,ATP1A1,BCL2,CAT,CNR2,CYP1A1,CYP2C9,CYP3A4,DNMT1,EDN1,ENPP1,ESR1,FN1,FOS,IFNG,IGF1,IL1B,JUN,KCNH2,LEP,NFKBIA,NOS1,NOS2,NOS3,NPPA,PDE4B,PTGS1,PTGS2,RNASE1,RXRA,SLC2A4,SLC6A4,TGFB1,THBS1,TNF,XDH | 84 (20) |
| 7 | MAPK8 | kinase | 8.49E-24 | ABCA1,ABCB1,AKT1,APOE,BCL2,CAT,CYP1A1,DNMT1,FOS,IFNG,IL1B,IL4,JUN,LEP,MAOB,MTHFR,MTTP,NOS2,NPPA,PPARD,PTGS2,TNF | 73 (22) |
| 8 | IL6 | cytokine | 1.41E-23 | ABCA1,ABCC2,ACVRL1,ADORA2B,AHR,AKT1,APOE,BCL2,CASP1,CD36,CYP1A1,CYP2C9,CYP3A4,DNMT1,ESR2,FN1,FOS,IFNG,IGF1,IL4,JUN,LEP,MPO,MTTP,NFKBIA,NOS2,NOS3,NR1I2,NR3C1,PON1,PTGS2,SLC2A4,TERT,TGFB1,THBS1,TNF | 79 (18) |
| 9 | RXRA | ligand-dependent nuclear receptor | 3.22E-23 | ABCA1,ABCB1,ABCC2,ABCG1,ADH1B,ADH1C,APOE,BRCA1,CAT,CD36,CETP,CYP2C9,CYP3A4,DRD2,FOS,IFNG,IL4,MAOB,MPO,NPPA,PPARD,RXRA,SLC2A4,TGFB1,TNF | 81 (20) |
| 10 | Ins1 | other | 7.91E-23 | ADH1C,AKT1,ATP1A1,CETP,CYP1A1,EDN1,FOS,IGF1,IL1B,IL4,JUN,LEP,MTTP,NOS1,NOS2,NOS3,NPPA,PGR,PPARD,PTGS2,RHOA,RXRA,SLC2A4,SLC6A2,SLC6A3,TNF | 87 (23) |
| 11 | AGT | growth factor | 2.98E-22 | ADH1C,ADORA2B,ATP1A1,BCL2,BRCA1,CAT,CAV3,CD36,CYP17A1,CYP2C9,EDN1,FN1,FOS,IGF1,IL1B,JUN,KCND3,LEP,NOS1,NOS2,NOS3,NPPA,PDE3A,PTGS1,PTGS2,RHOA,SLC6A2,TGFB1,TNF | 86 (23) |
| 12 | APP | other | 7.06E-22 | ABCA1,ABCG1,ABL1,ADORA3,AKT1,APOE,ATP1A3,BCL2,CAT,CHRM4,CHRNA7,DRD2,EDN1,ESR1,FN1,FOS,IFNG,IGF1,IL1B,IL4,JUN,KCND2,KCND3,MPO,NOS1,NOS2,NOS3,PDE4B,PPARD,PRKACA,PTGS2,RHOA,RYR3,TERT,TGFB1,TNF,TUBB | 91 (21) |
| 13 | IL1 | group | 8.22E-22 | ABL1,ADRB2,AHR,APOE,BCL2,CASP1,CYP17A1,CYP1A1,EDN1,FN1,FOS,IFNG,IGF1,IL1B,IL4,JUN,LEP,MTTP,NOS2,NR1I2,PTGS1,PTGS2,RXRA,TGFB1,TNF,XDH | 89 (23) |
| 14 | Mapk | group | 1.14E-21 | ABCB1,BCL2,CAT,ESR1,FOS,IFNG,IGF1,IL1B,JUN,LEP,NOS1,NOS2,NR3C1,PPARD,PTGS2,SLC6A2,SLC6A3,TNF | 87 (21) |
| 15 | NFkB (complex) | complex | 2.25E-21 | ABCB1,ABCG1,ADORA2B,AHR,APOE,BCL2,CAV3,CD36,CETP,CYP3A4,DRD2,EDN1,ENPP1,FN1,FOS,IFNG,IL1B,IL4,JUN,KCND3,NFKBIA,NOS1,NOS2,NPPA,PRKACA,PTGS2,SLC2A4,SOAT1,TERT,TGFB1,TNF,TPO | 73 (18) |
| 16 | LEP | growth factor | 4.14E-20 | APOH,BCL2,CD36,CYP17A1,DRD2,EDN1,ESR1,ESR2,FOS,IFNG,IGF1,IL1B,IL4,JUN,LEP,NOS1,NOS2,NOS3,NPPA,NR3C1,PGR,PTGS2,SLC2A4,TERT,TGFB1,THBS1,TNF | 85 (21) |
| 17 | HIF1A | transcription regulator | 1.13E-19 | ABCB1,ABCC8,ADORA2B,AKT1,APOE,BCL2,BRCA1,CD36,EDN1,FN1,FOS,IFNG,IGF1,IL1B,IL4,JUN,LEP,MGMT,NOS2,NOS3,PTGS2,SLC2A4,TERT,TGFB1,THBS1,TNF | 79 (20) |
| 18 | PI3K (complex) | complex | 1.14E-19 | ABCA1,ADRB2,AKT1,BCL2,CNR2,FN1,FOS,IFNG,IGF1,IL1B,IL4,JUN,LEP,NFKBIA,NOS2,NOS3,NPPA,PIK3CG,PTGS2,SLC6A3,SOAT1,THBS1,TNF | 80 (24) |
| 19 | RELA | transcription regulator | 2.28E-19 | ABCB1,AHR,APOE,BCL2,CYP17A1,CYP1A1,CYP2C9,CYP3A4,EDN1,FN1,FOS,IFNG,IGF1,IL1B,IL4,JUN,MGMT,NFKBIA,NOS2,PDE4B,PTGS2,SLC2A4,TERT,TGFB1,TNF,TPO | 79 (19) |
| 20 | IGF1 | growth factor | 2.34E-19 | ABCA1,ADORA2B,AKT1,BCL2,BRCA1,CYP17A1,EDN1,ESR1,ESR2,FN1,FOS,IGF1,IL1B,IL4,JUN,LEP,NFKBIA,NOS2,NPPA,NR3C1,PDE3A,PGR,PTGS2,SLC2A4,TGFB1,THBS1,TNF | 78 (22) |


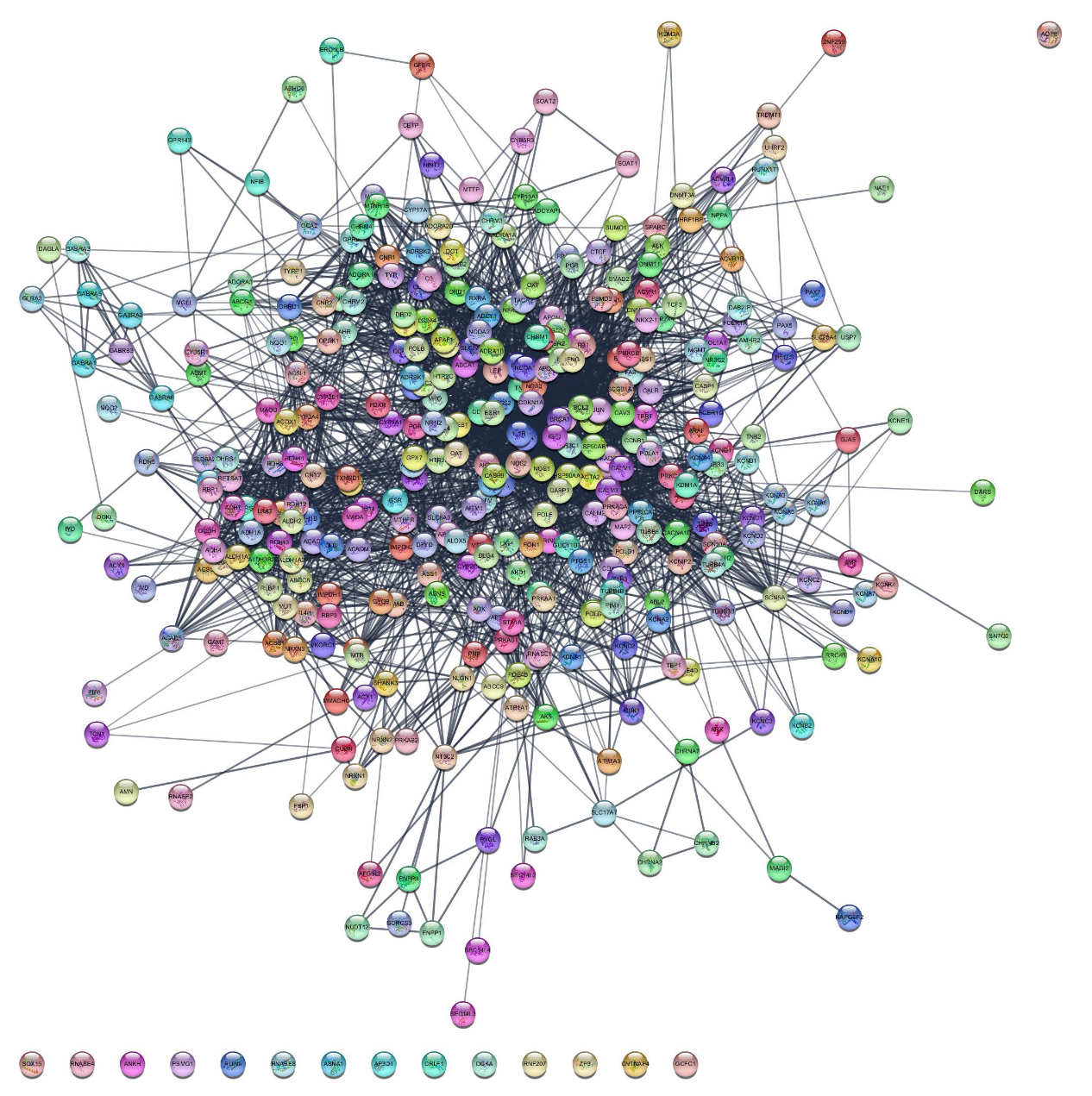


**Fig. 1. Identification of targets of SMI.** SMI targets PPI network. Nodes represent proteins. Edges represent protein-protein associations and line thickness indicates the strength of data support.


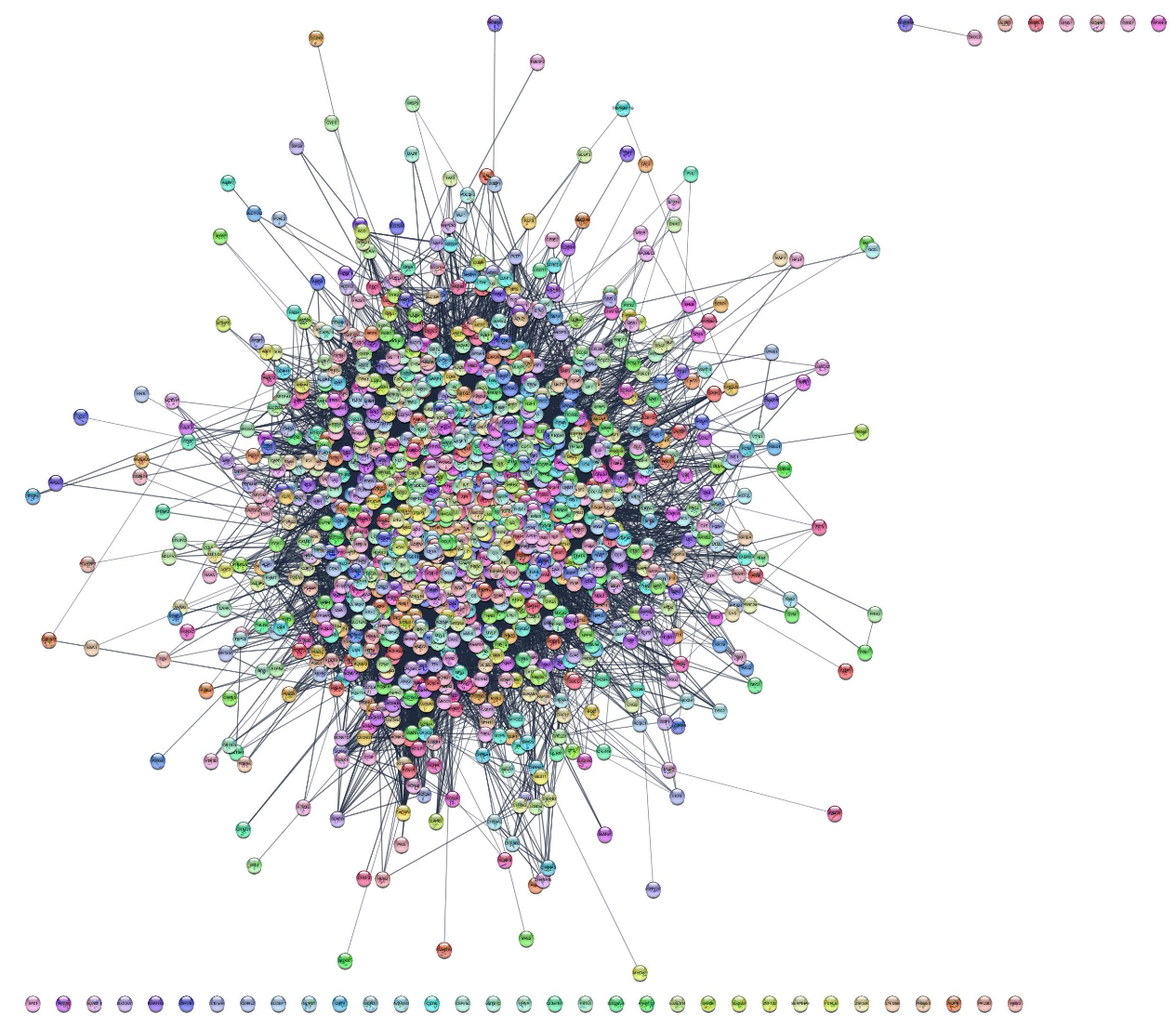


**Fig. 2. Identification of targets of CVD.** CVD targets PPI network. Nodes represent proteins. Edges represent protein-protein associations and line thickness indicates the strength of data support.
